# Disulphide and sequence-encoded conformational priors guide nanobody structure prediction

**DOI:** 10.64898/2026.02.13.705647

**Published:** 2026-02-13

**Authors:** Montader Ali, Mateusz Jaskolowski, Matthew Greenig, Tung H. Nguyen, Mia Crnogaj, Eva Smorodina, Aubin Ramon, Haowen Zhao, Monica L. Fernández-Quintero, Elodie Ghedin, Victor Greiff, Pietro Sormanni

**Affiliations:** 1 Yusuf Hamied Department of Chemistry, University of Cambridge, Cambridge, UK; 2 Department of Pharmacology, University of Cambridge, Cambridge, UK; 3 Department of Chemical Engineering, Imperial College London, South Kensington Campus, London, UK; 4 Department of Immunology, University of Oslo and Oslo University Hospital, Oslo, Norway; 5 Systems Genomics Section, Laboratory of Parasitic Diseases, National Institute of Allergy and Infectious Diseases (NIAID), National Institutes of Health (NIH), Bethesda, Maryland 20894, USA; 6 Department of Integrative Structural and Computational Biology, The Scripps Research Institute, La Jolla, CA 92037, USA

## Abstract

Nanobody binding is largely governed by the HCDR3 loop, which adopts distinct placement regimes relative to the framework: compact, framework-contacting (kinked blueprint) and solvent-exposed (extended blueprint). Many nanobodies also contain additional cysteines that form non-canonical disulphide bonds, imposing covalent constraints on binding-loop conformations. Current structure predictors are typically trained and benchmarked with smooth coordinate-based objectives, so models may appear reasonable under root-mean-square deviation (RMSD), while adopting an incorrect HCDR3 blueprint or failing to recover the native disulphide connectivity, impacting paratope geometry and functional interpretation. Here, we show that the HCDR3 blueprint is predictable from sequence alone, allowing for explicit constraints during modelling. We implement these principles into NbForge, a lightweight nanobody folding model that incorporates blueprint- and disulphide-aware inductive biases and is trained with filtered self-distillation. NbForge improves recovery of HCDR3 blueprint and non-canonical disulphide formation over previous lightweight models and achieves coordinate accuracy at par to state-of-the-art, large, resource-intensive predictors, while running at sub-second inference speed. We show that using NbForge monomer models as templates further improves the success rate of predicting nanobody-antigen complexes. Together, these results motivate blueprint- and disulphide-aware benchmarks for nanobody modelling beyond RMSD, and show that appropriate inductive biases can close the performance gap to heavyweight predictors. We make the sequence classifier (NbFrame) and NbForge available for download and via a user-friendly web server.

## 1 Introduction

Nanobodies ($V_{HH}s$) are single-domain antibodies naturally produced in camelids [1]. These molecules are becoming increasingly recognised as a promising molecular modality for a wide range of therapeutic applications following the approval of the first nanobody drug in 2019 [2, 3]. Beyond monovalent binding, $V_{HH}s$ are genetically encodable, modular domains that can be assembled into multivalent or multispecific formats through straightforward head-to-tail fusion (typically via flexible peptide linkers), enabling compact multi-target binders and avidity engineering [4, 5]. Their small size and high stability also make them attractive targeting ligands for engineered delivery platforms (including viral nanoparticles/virus-like particles and lipid nanoparticles for nucleic-acid delivery), and as antigen-recognition domains in engineered cell therapies such as $V_{HH}$-based CAR-T cells [6, 7, 8, 9]. Additionally, the compact structure of nanobodies can readily be tuned for high affinity and specificity, while often retaining favourable stability and manufacturability profiles [10, 11, 12].

Deep-learning protein structure prediction has made high-throughput modelling of antibody variable domains practical, enabling structure-informed decision making earlier in discovery or engineering pipelines [13, 14, 15, 16]. In current antibody and nanobody workflows, structural models are used to triage and cluster repertoire or library hits, prioritize candidates via paratope–epitope prediction ranking, design focused mutational panels for affinity maturation, or flag developability risks (e.g., instability, aggregation propensity, and chemical liabilities) before committing to extensive experiments [17, 18, 19, 20, 21]. Across these structure-dependent applications, the key limitation is increasingly not whether a nanobody structural model can be generated, but whether it is sufficiently accurate in the regions that matter most for function – particularly the hypervariable binding loops, whose sequence and conformational diversity remains a persistent challenge for structure prediction. [22, 23, 24].

Structurally, nanobodies are single immunoglobulin variable domains in which a rather conserved β-sandwich framework (FR) supports three hypervariable loops, referred to as heavy-chain complementarity determining regions (HCDR1–3), that structurally define the binding region and contain most of the antigen-contacting residues (i.e., the paratope) [25]. A defining structural tendency of $V_{HH}$ repertoires is a HCDR3 loop that is longer on average than that of conventional antibodies. This loop is also the most variable, and it frequently contributes the biggest share of antigen contacts [26], expanding molecular reach and enabling engagement of recessed or concave epitopes, but also enlarging the conformational space that structure prediction must resolve[24]. Unlike conventional antibody $V_{H}$ domains, nanobody $V_{HH}s$ are devoid of a paired light (L) chain [27, 28]. Consequently, the former $V_{H}-V_{L}$ interface remains solvent-exposed in $V_{HH}s$ and can serve as a stabilizing framework surface onto which HCDR3 folds back in kinked conformations. Consistent with this, $V_{HH}$ frameworks exhibit adaptations at the former interface (notably increased hydrophilicity in FR2), and the absence of $V_{L}$ reshapes the energetic balance of loop packing versus solvent exposure [29, 30, 28]. In practice, this architectural difference reduces the size of the molecule and often introduces couplings between the framework sequence and HCDR3 loop placement, such that framework regions corresponding to the former $V_{H}/V_{L}$ interface (including FR2) can form contacts with HCDR3 residues, and in some cases also mediate contact with the antigen [24].

**Figure 1:**
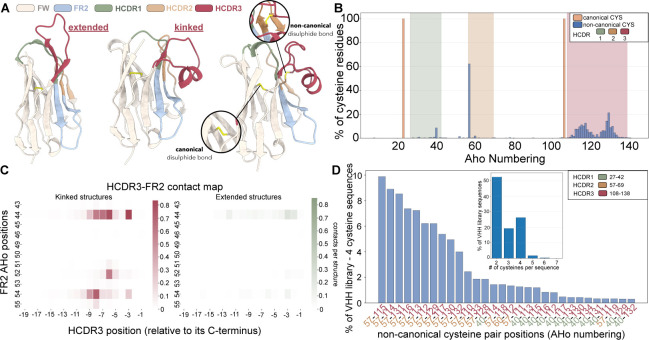
Structural and sequence features associated with kinked versus extended $V_{HH}$ HCDR3 conformations. (**A**) Representative $V_{HH}$ structures with an extended HCDR3 (left) and a kinked HCDR3 (centre and right). Framework (FW), framework region 2 (FR2), and HCDR1–3 are colour-coded (legend); the canonical intradomain disulphide bond is indicated and an example non-canonical disulphide involving HCDR3 is highlighted (Inset). (**B**) Distribution of cysteine residues of $V_{HH}$ sequences in AHo numbering from a $V_{HH}$ library of sequences [36], separating canonical and non-canonical cysteines; shaded bands denote HCDR1–3 positions. (**C**) Mean HCDR3–FR2 contact maps for kinked, *n* = 481 (left), and extended, *n* = 346 (right), $V_{HH}$ PDB structures (labelled by NbFrame), with HCDR3 indexed relative to its C terminus and FR2 positions shown in AHo numbering. (**D**) Frequencies of non-canonical cysteine-pair positions (AHo numbering) among $V_{HH}$ library sequences [36] containing four cysteines; inset shows the distribution of total cysteine counts per sequence across the whole library.

This coupling between framework and HCDR3 anchoring is a key determinant that structure-prediction models must capture to effectively facilitate nanobody discovery and engineering. Experimentally solved $V_{HH}$ structures populate two broad, qualitatively distinct HCDR3 placement blueprints that differ in loop positioning and in the extent of loop–framework contacts – extended loops with no or very limited FR engagement, and kinked loops with varying amounts of FR engagement, respectively (Figure 1, Table 7) [29]. In the extended regime, HCDR3 typically projects outward into the solvent, expanding molecular reach and enabling access to recessed epitopes. In the kinked regime, HCDR3 folds back onto the domain, packing against the framework surface and creating a paratope shaped by non-local loop–framework contacts. A kinked-to-extended swap is not a small geometric drift, but a transition between alternative packing solutions that strongly affects the paratope conformation, leading to different binding-interface geometries. Therefore, correct HCDR3 blueprint prediction has important implications for downstream tasks, including nanobody-antigen docking, paratope and epitope prediction, and mutation design.

The conformational diversity of HCDR3 reflects, in part, how $V_{HH}$ framework features shape loop anchoring against the domain versus projection into solvent [24, 31]. While the blueprint conformations have been assigned from experimentally solved structures using geometric or contact-based criteria, structure-based definitions are not directly usable at the point where they are most needed: when reasoning about large sets of sequences that do not yet have structures (e.g., during library assessment, developability screening, or dataset construction for model training and benchmarking). In these sequence-only, discovery-scale settings, candidate triage often relies on readily computable features such as HCDR3 length distributions or cysteine motifs, which do not explicitly encode the global placement of HCDR3 relative to the framework [32, 33]. Taken together, these considerations motivate the need for a sequence-level, $V_{HH}$-specific prior over the HCDR3 blueprints.

A second, largely orthogonal determinant of nanobody paratope arrangement is the presence of additional, non-canonical intradomain disulphide bonds. Beyond the conserved Ig-domain disulphide, a substantial fraction of camelid $V_{HH}s$ carry extra cysteines that form cross-links most commonly tethering HCDR3 to HCDR2, but also linking HCDR3 to HCDR1 or other framework positions near the former $V_{H}-V_{L}$ interface (e.g., FR2), and in some cases forming intra-HCDR3 disulphides (Figure 1, Table 2) [34, 31, 12]. These bonds are frequently discussed as evolutionary and engineering solutions to the expanded conformational freedom introduced by long HCDR3 loops; analysis of the llama wild-type $V_{HH}$ repertoire shows that over 25% of sequences contain non-canonical disulphide bonds involving CDR3 cysteines and germline-encoded cysteines at variable positions, indicating a camelid-specific mechanism of conformational preselection and stabilization that expands paratope geometry and antigen-binding diversity [35]. Indeed, by covalently constraining loop topology, they can rigidify and pre-organize binding-competent conformations, modulate loop–framework packing, and often increase structural robustness (with context-dependent effects on affinity and stability) [12, 31, 30]. Accurately capturing non-canonical disulphide bridges is also directly relevant to developability and engineering decisions. Empirically, these bonds are often coupled to thermal stability, refolding behaviour, and aggregation propensity [30, 31]. In practical discovery settings, accurate prediction of disulphide constrained conformations is important not only for structural interpretation, but also for prioritizing sequences whose folds are likely to be stable and functionally deployable.

Taken together, the kinked/extended blueprints and non-canonical disulphide bridges highlight that nanobody loop modelling is not purely a continuous optimization problem over backbone coordinates. Instead, it is a mixed inference problem: the structure must satisfy a small number of discrete features (HCDR3 blueprint and disulphide-bridge formation), while simultaneously resolving the continuous degrees of freedom within these constraints. This observation motivates approaches that incorporate sequence-level priors over HCDR3 placement and explicit geometric objectives for disulphide-constrained topologies, as well as benchmarks that interrogate these discrete commitments directly, especially in the long-loop, cysteine-rich blueprints consequential for nanobody engineering.

We therefore introduce NbFrame, a lightweight classifier that calculates the probability of the HCDR3 adopting a kinked or extended blueprint directly from sequence, and NbForge, a lightweight, all-atom $V_{HH}$ structure prediction framework that directly incorporates nanobody-relevant features into both training and evaluation. NbForge is trained using three complementary strategies: (i) we expand structural supervision by leveraging variable heavy chains from conventional Fv antibodies alongside curated $V_{HH}$ structures to better cover the HCDR3 length and topology distribution; (ii) we introduce an explicit disulphide-geometry objective to encourage physically plausible non-canonical disulphide formation (including SG–SG and Cβ−Cβ distance terms, and associated angular/dihedral constraints); and (iii) we scale learning through filtered self-distillation on a large naïve $V_{HH}$ sequence library, folding only high-confidence kinked and extended sequences and retaining only predictions that satisfy disulphide topology, avoid bond violations, and remain consistent with NbFrame-implied HCDR3 placement. The resulting synthetic set is then used to train the model and is complemented by final training/fine-tuning on experimentally solved variable-heavy chains, yielding a fast, HCDR3 blueprint and disulphide bridge constrained predictor designed for discovery-scale use.

## 2 Results

### Data curation as a prerequisite for nanobody-specific learning

A central practical constraint in nanobody structure prediction is that the available set of experimentally solved $V_{HH}$ structures remains small and heterogeneous in quality (Table 2). We therefore began by constructing a training set from the Structural Antibody Database (SAbDab) [37], which explicitly stratifies the nanobodies by HCDR3 length, kinked and extended, as well as the presence of a non-canonical disulphide bridge. Starting from antibody-containing PDB entries, we separated conventional antibodies from nanobodies, isolated each variable heavy chain as a standalone chain-level sample (both $V_{H}$ and $V_{HH}$), and then removed structures containing missing residues within the heavy-chain core. Missing internal residues introduce silent label noise where the model is asked to learn a fold that is not fully specified by the coordinates, and downstream uncertainty estimate scores can be falsely attributed to loop modelling accuracy rather than incomplete supervision [38]. This procedure resulted in a consistent set of structures of antibody heavy chain variable domains ($V_{HH}:N_{\mathrm{sequences}}=892,\;N_{\mathrm{structures}}=3128,\;V_{H}:N_{\mathrm{sequences}}=3672,\;N_{\mathrm{structures}}=13,261$).

### Development of NbFrame as a structure- and sequence-based HCDR3 classifier

Given the functional importance of HCDR3 conformational blueprint, we sought to identify structural features that distinguish kinked from extended conformations in $V_{HH}$ domains. A comprehensive feature set capturing multiple aspects of the conformational geometry surrounding the HCDR3 and the FR2 regions was used to classify the conformations of the PDB structures. We selected six features based on biological rationale and building on previous studies: backbone angles at both the N-terminal ($\alpha_{N},\;\tau_{N}$) and C-terminal ($\alpha_{C},\;\tau_{C}$) stems of the HCDR3, contact density between HCDR3 and FR2 (number of atomic contacts normalized by HCDR3 length), and relative solvent accessibility (RSA) of key FR2 hydrophobic positions (AHo positions 44 and 54) [29, 26].

To establish ground-truth labels, we performed Gaussian Mixture Model (GMM) clustering in the six-dimensional feature space, revealing two geometrically well-separated clusters (Figure S2; see Methods). We sampled 50 structures from each cluster, including both cluster cores and boundary cases, and classified each by manual inspection. This yielded 60 extended and 40 kinked labels. The imbalance reflects that GMM clustering captured geometric patterns primarily linked to angles, whereas the structural definition of kinked conformations additionally requires physical contacts with FR2 (see Methods). We trained a logistic regression classifier on these 100 manually labelled structures, representing each input with the six features described above. The model achieved ROC-AUC of 0.99 and accuracy of 94.0% on the held-out test set (n=100) (Figure S3). Feature coefficients revealed that contact density (+1.86) and FR2 RSA (−1.16) were the dominant predictors (Figure S4), consistent with the functional role of kinked conformations in shielding FR2 hydrophobic residues.

As kinked and extended HCDR3 conformations are associated with characteristic framework sequence patterns, we identified framework positions where specific amino acids showed statistically significant enrichment in kinked versus extended structures (Fisher’s exact test, *p* < 0.05, minimum 30 observations). From these candidates, we selected 20 hallmark features with the strongest discriminative signal at biologically relevant positions (Figure S5): FR1 (positions 12, 15, 17), FR2 (positions 44, 51, 54, 56), and FR3 (positions 85, 103, 107). Position 107, adjacent to the conserved Cys106 at the HCDR3 junction, contributed multiple hallmarks, while positions 44 and 54 correspond to the classic FR2 hydrophobic residues that interact with kinked HCDR3 loops (Figure 1). Each sequence was encoded by the log_2_ fold-change enrichment value of each of these 20 hallmarks, when the sequence contained that specific amino acid at that position (Table 7).

We trained a logistic regression model from the sequences of 829 labelled structures, deliberately restricting features to framework positions to avoid potential bias from hypervariable CDR sequences or unusual CDR motifs found in engineered or synthetic $V_{HH}s$. Evaluated on the same 100 held-out structures with manually validated labels, the sequence classifier achieved ROC-AUC of 0.94 and accuracy of 86.0% (Table 1, Figure S3 and S9).

### Building a self-distillation dataset of nanobody structures from filtered immune repertoires

**Figure 2:**
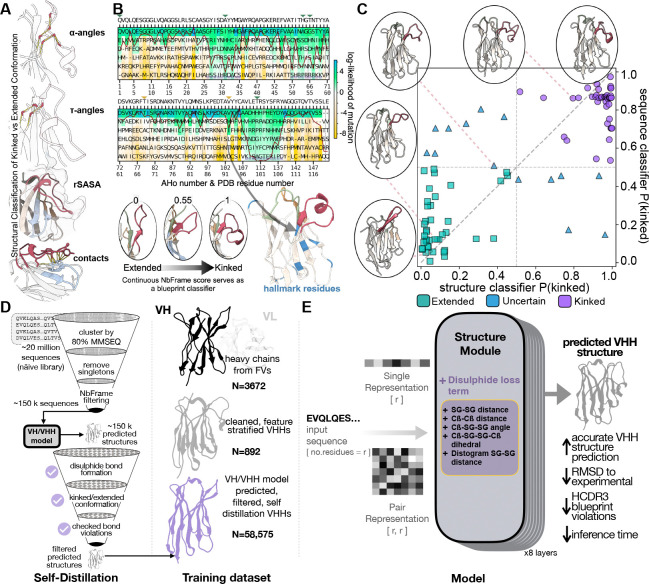
NbFrame structure and sequence classifier, creation of the self-distillation dataset, and architecture of the NbForge model. **(A)** Structural definition of the kinked/extended HCDR3 blueprints using geometric descriptors; these features provide a continuous NbFrame score that classifies blueprint states. **(B)** Sequence-based classification using framework hallmark residues via log fold-change values of kinked vs extended blueprints (see Table 7) to provide a kinked probability score. **(C)** Agreement between sequence-based and structure-based NbFrame classifier scores-with extended, kinked, and ambiguous cases shown with representative structures. **(D)** Overview of the NbForge $V_{HH}$ self-distillation sequence and structure filtering steps. Sequences from the $V_{HH}$ library are first clustered by MMSEQ2 at 80% similarity (the first sequence from each cluster is taken); sequences that were not clustered (singletons) were filtered out. NbFrame’s sequence classifier was used to remove sequences classified as uncertain. A $V_{H}/V_{HH}$ trained model (see Methods) folded the filtered sequences. These predicted structures were then filtered for structural violations, incongruent HCDR3 blueprint, or missed expected NCDB, and the remaining structures were used to train the final model together with the experimental $V_{H}$ and $V_{HH}$ structures. **(E)** NbForge architecture for unbound $V_{HH}$ structure prediction. During the forward pass (training and inference), the input sequence is embedded into per-residue (‘single’/node) features, and residue-residue (‘pair’/edge) features are initialised with relative positional encodings (L×L). Single and pair features are then iteratively refined within an invariant point attention (IPA)-based geometry module (AF2 structure-module-style update stack; same IPA-based architecture family used in NanoBodyBuilder2), which updates residue rigid frames to produce 3D coordinates and per-residue confidence (pLDDT head not shown). An additional ‘disulphide objective’ loss term is used during training to favour physically valid disulphide bridges.”

$V_{HH}$ repertoires span an enormous sequence space (often quoted as ~ 10^12^–10^15^ possible antibodies), and the long HCDR3 loops that are common in single-domain antibodies further amplify conformational diversity and consequent modelling uncertainty [22, 39, 40, 41]. Experimentally determined nanobody structures sample only a small fraction of this diversity (892 unique sequences in our curated set), limiting the sequence–structure coverage that can be learned directly from ground-truth data. To expand effective coverage while retaining modelling fidelity, we therefore adopted a self-distillation strategy: starting from a large naïve $V_{HH}$ sequence collection (22 million sequences) [36], we applied two sequential filters to enrich for sequence diversity while favouring sequence features and structural filters consistent with accurate structure prediction (Figure 2D).

First, we clustered sequences using MMseqs [42] at 80% full-sequence identity and removed singletons, yielding approximately 650,000 non-redundant sequences. Second, to focus specifically on sequences with clear HCDR3 conformational blueprint markers, we retained only those predicted with high confidence to be kinked (NbFrame score 0.99) or extended (NbFrame score 0.20), intentionally removing ambiguous intermediates. Although this filtering step narrows the scope of the synthetic set, it provides a principled way to discard low-confidence models that violate the expected structural blueprint, yielding a higher-quality set for downstream training. After this stage, we obtained 154,115 sequences, which were folded at scale with a $V_{H}/V_{HH}$-trained version of the model (Table 2; see Methods). Briefly, this $V_{H}/V_{HH}$ teacher model is a lightweight, structure-module–only predictor derived from ABodyBuilder3/AlphaFold2; it retains the AlphaFold2 structure module and pLDDT head, rather than the full Evoformer/MSA/template pipeline (see Methods; Figure 2E). We augment the base coordinate objectives with an explicit disulphide-bridge loss that supervises cysteine pairing and geometry.

To reduce the occurrence of inaccuracies, which can later bias the training when such modelled structures are used for self-distillation, we applied a stringent, nanobody-specific filtering strategy. First, we removed predictions with mispaired disulphide bonds (see Methods). Second, we filtered out structures whose predicted HCDR3 blueprint was inconsistent with the NbFrame-evaluated blueprint based on the structure classifier (Table 1, Figure 2, see (Methods). Furthermore, we applied TopModel (amide planar bond) [43] and peptide bond filtering that removed structures with backbone violations. Finally, we required a minimum mean HCDR3 pLDDT (0.7) as a coarse confidence criterion, ensuring that retained structures were not only topologically plausible but also internally self-consistent under the model’s own uncertainty estimates.

In summary, this curation strategy yields a synthetic self-distillation set comprising 58,575 structural models, enriched for sequences with clear kinked/extended commitments and for predictions that satisfy disulphide-bridge formed structures with loops whose conformation is predicted with high pLDDT confidence. These models are generated using a $V_{H}/V_{HH}$-trained NbForge teacher check-point (the same lightweight AlphaFold2 structure-module + pLDDT architecture, augmented with our disulphide-geometry objective- see Methods) trained on the curated set of experimentally solved $V_{H}$ and $V_{HH}$ domains. We use this teacher to fold the 154,115 blueprint-confident library sequences at scale, then retain only predictions that pass the NCDB-specific topology, blueprint-consistency, and bond-violation filters described above and in [44]. The resulting 58,575-model set provides synthetic supervision for self-distillation: we retrain the NbForge architecture under blueprint and disulphide constraints on these filtered models, and then fine-tune on experimentally solved heavy chains to obtain the final predictor.

### Dataset composition and benchmark design

To contextualise the conformations represented in our training, synthetic, and evaluation data, we summarise key HCDR3 and cysteine-linked properties across datasets in (Table 2). Experimental $V_{HH}$ structures in SAbDab exhibit a mean HCDR3 length of approximately 14 residues (14.7±4.5 for all chains, 14.4±4.3 after deduplication), with non-canonical disulphide bonds present in a minority of cases (17.0% and 13.9%, respectively) and a strong skew toward kinked conformations (69.6% and 68.6%). In contrast, the naïve 22 million $V_{HH}$ sequence library we use to approximate repertoire-scale diversity is shifted toward both longer loops (15.2±4.5) and a higher prevalence of non-canonical cysteine patterns (26.3%), with a modest reduction in kinked frequency (61.8% labelled via NbFrame).

We therefore designed our held-out evaluation test set of $V_{HH}$ structures to reflect these statistics. The NanobodyBuilder2 (NBB2) test set closely mirrors SAbDab in mean HCDR3 length (14.0±4.5) but contains very few non-canonical disulphide bonds (2.5%) and is strongly enriched for kinked HCDR3s (80.0%), limiting its ability to stress test disulphide-constrained loop topologies and a balanced blueprint conformation. By contrast, our NbForge test set is deliberately shifted toward longer HCDR3 loops (17.8±4.3) and substantially higher non-canonical disulphide prevalence (31.3%), while maintaining a more balanced kinked/extended composition (62.5% kinked) and with most sequences displaying up to 50% HCDR3 residue differences to the training set (Figure S1). Moreover, we ensured that all of our test-set structures were released after December 2022 (past the AF3/Boltz1 cut-off date of September 2021 and that of NBB2 of July 2021), which - together with the high diversity from the training set (i.e., from all other $V_{HH}$ structures) - also makes it a good benchmark set for these modelling tools. To ensure no data-leakage, we removed PDBs with homologous sequences to this test set, and show that our test set’s HCDR3 sequences are at most ~ 60% similar to the training/validation set (Figure S1). This test-set design enables evaluation not only of coordinate accuracy, but also of whether models recover the structural features that determine the $V_{HH}$ loop conformation - which are under-represented in standard benchmarks (HCDR3 blueprints and non-canonical disulphide bridges).

### Coordinate accuracy on the NbForge test set

NbForge is built as a lightweight model using only the structure module and pLDDT module from AlphaFold2 (see Methods, Figure 2D). The structure-module focused architecture for a nanobody folding model was initially used by the NanoBodyBuilder2 (NBB2) model, which displayed competitive RMSDs against AlphaFold2 nanobody structure prediction, at a fraction of the inference time [45], and is widely used in $V_{HH}$ discovery campaigns [46]. Larger diffusion-based models have since shown to be the more accurate models in predicting $V_{HH}s$ backbones [23, 47, 24]; these large models rely on sophisticated architectures, that demand extensive GPU resources, MSA inputs, with some models also accepting template structures, and are substantially more costly in terms of inference time (Table 3). We therefore evaluate our NbForge model against the lightweight NBB2 model, as well as the diffusion-based AF3 (best-in-class) and Boltz1 heavy models (open-source). Boltz2 and other recently released models could not be compared due to large overlap between their training sets and our test set, but Boltz2 has already been shown to perform similarly to Boltz1 on $V_{HH}S$ [48].

Because each model is evaluated on each $V_{HH}$ in the same test set, we report (i) an overall repeated-measures test for model effects (Friedman) and (ii) planned paired post hoc comparisons (Wilcoxon signed-rank) focusing on NbForge versus the other models. NbForge improves significantly over NBB2 for both all-backbone RMSD and HCDR3 RMSD (*p* < 0.001 paired Wilcoxon for both-Table S1), with large paired effect sizes (rank-biserial correlation magnitudes $\left\|r_{rb}\right\|\approx 0.65$ and $\left|r_{rb}\right|\approx 0.63$). Against the diffusion-based large models, NbForge is competitive on HCDR3: median HCDR3 RMSD is 2.95 Å for NbForge versus 2.95 Å for AF3 and 3.39 Å for Boltz1 (all 95% CIs overlap), and these differences are not statistically distinguishable under paired testing (NbForge vs AF3: *p* = 0.49; NbForge vs Boltz1: *p* = 0.90). However, AF3 and Boltz1 show significantly lower framework median-RMSD (0.70 Å and 0.65 Å versus 0.72 Å) (Table 3,S1), suggesting that while NbForge matches the large model’s performance on the most variable loop region, some global framework accuracy remains achievable through broader priors or MSAs, for example. Notably, in nanobodies the dominant sources of functional specificity and modelling uncertainty are concentrated in the hypervariable CDR loops, especially HCDR3, so parity in HCDR3 loop accuracy and constraint satisfaction is typically more consequential for downstream utility and design than sub-Å differences in the comparatively rigid framework.

### HCDR3 kinked/extended blueprint recovery

We next evaluated whether each method recovers the correct global HCDR3 blueprints by matching each predicted structure to its corresponding experimental chain and comparing the loop placement using NbFrame’s structural classifier definition (see Methods). All four models achieve high conformation recovery on this benchmark (Table 3), with AF3 performing best at 44/47 (93.6%), NbForge recovering 43/47 (91.6%), followed by Boltz1 at 42/47 (89.4%), and finally NBB2 at 38/47 (80.8%) (Figure 4A). The overall near-ceiling performance across models suggests that the kinked/extended distinction is strongly encoded by sequence features and can be learned reliably even from comparatively limited $V_{HH}$ structural data, supported by the high PR-AUC of the NbFrame sequence classifier, which was built entirely from residue frequencies.

**Figure 3:**
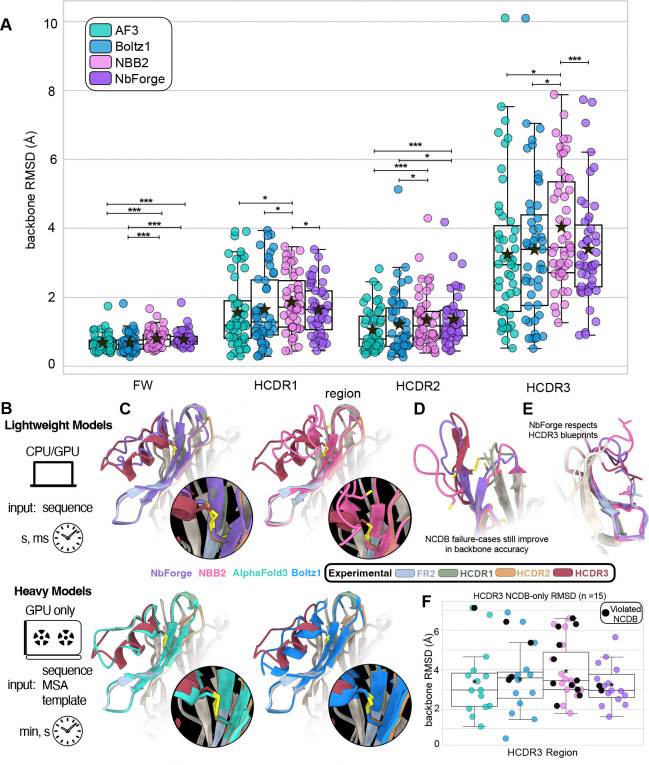
Nanobody structure prediction accuracy, HCDR3 blueprint fidelity, and NCDB formation. **(A)** Swarmplot of full-backbone RMSD to experimental structures in the held-out test set (*n* = 47) calculated following framework-region superimposition across different $V_{HH}$ regions (x-axis) for AlphaFold3 (AF3), Boltz1, NBB2, and NbForge (legend). Boxes represent the first and third quartiles of the distribution, whiskers represent the 1.5 interquartile range, the horizontal line is the median and the star marker the average. Brackets denote Wilcoxon pairwise statistical comparison, where *, **, and *** denote *p* < 0.05, *p* < 0.01, and *p* < 0.001, respectively; see Table S1 for details. **(B)** Inference models compared in this work: “lightweight” single-sequence predictors versus “heavy” predictors. **(C)** Representative example (PDB: 9g1y) to highlight the influence of correct disulphide-bridge prediction on backbone accuracy, with close-ups highlighting loop placement and disulphide geometry. **(D)** Case study (PDB: 8z8v) highlighting NCDB-recovery failure cases that still show improved backbone accuracy among lightweight models (NbForge vs NBB2). **(E)** Case study showing that NbForge recapitulates experimental HCDR3 kinked-blueprint contacts. **(F)** Like A but only for the HCDR3-region of the NCDB-containing subset (*n* = 15), with models violating the non-canonical disulphide constraint indicated in black; values are reported in Table 4.

**Figure 4:**
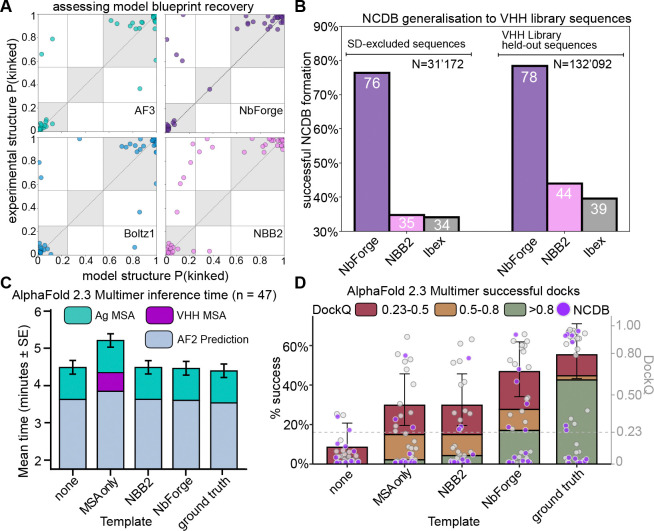
HCDR3 blueprint agreement, non-canonical disulphide (NCDB) recovery, and downstream docking performance. (**A**) Agreement between model-predicted and experimentally observed HCDR3 blueprint states, shown as structure-classifier probabilities P(kinked) for experimental structures (y-axis) versus predicted structures (x-axis) from AlphaFold3 (AF3), Boltz1, NbForge, and NanobodyBuilder2 (NBB2). Points represent individual nanobodies from the *n* = 47 test set (see also Table 3). (**B**) Bar plot reporting the fraction of sequences forming the expected NCDB for the lightweight models NbForge, NBB2, and Ibex on two different large sequence datasets; N indicates dataset size; values within bars indicate percentages. Both datasets contain only sequences with 4 or 5 cysteines, the two forming the canonical disulphide (AHo positions 23 and 106), one in the HCDR3 and another at either AHo positions 57 or 40. See Methods for more information. (**C**) and (**D**): AF2M inference time (C) and DockQ-based performance (D) in modelling $V_{HH}$-antigen complexes under different input conditions, including inputting monomeric templates predicted by NbForge or NBB2 (x-axis). In D, the left axis (bars) is the cumulative percent of structural models in each DockQ-accuracy group (legend), and the right axis (data points) is the DockQ score of each modelled structure. The “ground truth” group is obtained by providing to AF2M as template the monomeric nanobody structure as found in the experimentally determined bound complex, and it thus serves as a positive control for the highest achievable performance via templated predictions.

Despite this high performance, the remaining errors are informative. Across models, misclassifications are dominated by missed kinked blueprints (false negatives), i.e., predicting an extended blueprint when the experimental structure exhibits framework contacts characteristic of a kinked blueprint. This trend is most pronounced for NBB2, where some violations with the experimental structure’s kinked blueprint are recovered by the other models (Figure 3E, 4). These examples underscore that, while blueprint recovery is picked up by the models implicitly, the structure module on its own with only backbone coordinate loss terms (the main head of the NBB2 architecture) can still fail to capture subtle loop-framework packing patterns that the larger models recover more consistently.

### Non-canonical disulphide bridge recovery

Recovery of non-canonical disulphide bridges separates methods more strongly than kinked/extended blueprints (Table 3). AF3 recovers 14/15 (93.3%), NbForge recovers 13/15 (86.7%), and Boltz1 recovers 11/15 (73.3%), whereas NBB2 recovers only 4/15 (26.7% - exact McNemar p-value vs NbForge 0.004). The failure pattern in NBB2 is qualitatively consistent with a HCDR3 misplacement: many of the NCDB cases where NBB2 fails are ones that the other models recover (Figure 3F), and these same targets correspond to some of NbForge’s most accurate HCDR3 predictions (Table 3, 4), suggesting that failing to satisfy the covalent constraint often coincides with broader loop misplacement. At the other extreme, one NCDB target (Figure 3D) is missed by all models, and is also among the highest-error HCDR3 cases for the strongest predictors (PDB ID: 8zer). Notably, this NCDB corresponds to cysteines that are in an unorthodox position of typical NCDB cysteine positions in $V_{HH}$ AHo sequence highlighted in Figure 1 (position 32 and 111 respectively).

Next, we stratified RMSD by NCDB status. As expected, the NCDB-bearing subset is enriched for substantially longer HCDR3s (mean 19.6 residues versus 13.9 residues in the non-NCDB subset), reflecting a different region of sequence and conformational space than most HCDR loops present in standard $V_{HH}$ benchmarks. Despite the longer loop, NbForge maintains strong HCDR3 accuracy on NCDB-bearing targets and remains among the top-performing methods in this subgroup (median HCDR3 RMSD 2.95 Å for NbForge, compared with 2.97 Å for AF3, 3.58 Å for Boltz1, and 3.45 Å for NBB2; Table 4, Figure 3). Boltz1 exhibits a modest performance degradation in median HCDR3 RMSD on NCDB-bearing targets relative to the non-NCDB subset (2.92 → 3.58 Å). NBB2, unlike the other models, consistently performs worse on both classes of NCDB and no NCDB cases (3.45 and 3.74 Å respectively), whereas NbForge’s and AF3’s performances remain sub 3 Å between NCDB and no-NCDB structures (NbForge: 2.95 → 2.95 Å and AF3: 2.75 → 2.97 Å). This pattern is consistent with the intended role of the explicit disulphide-aware objective, where within a covalently constrained loop, correctly recovering the disulphide bridge can reduce the effective loop conformational space and stabilize loop placement (Figure 3C, D, F).

### Inference time

Prediction time is a key feature of folding models, as faster inference time can enable large-scale dataset evaluation of candidates from design campaigns or laboratory library-screening without incurring prohibitive compute costs. We measured end-to-end wall-clock inference time on the 47-target test set (Table 5). While AlphaFold3 and Boltz1 require on the order of tens of seconds per structure on GPU (~ 1 minute with MSA, ~ 40 s without MSA), NbForge predicts structures in less than a second per sequence on both CPU and GPU (Table 5). This throughput difference, combined with NbForge’s competitive HCDR3 RMSD, strong blueprint recovery and NCDB formation, is decisive to enable screening-scale $V_{HH}$ structure prediction in discovery workflows where thousands to millions of candidates may be triaged.

### Implications for nanobody design and discovery

In library triage or de novo nanobody generation, misplacing a disulphide-constrained HCDR3 can yield a fold that appears geometrically plausible yet is fundamentally incompatible with the relevant paratope conformation. To address whether NbForge can correctly predict NCDBs at library scale, we compared NbForge against two lightweight models that offer comparable speed: NBB2 and Ibex, a recently introduced nanobody predictor trained on public structural data [48]. We predicted structures for two stringent sequence sets that were excluded from our main training and self-distillation pipelines: (i) self-distillation-excluded sequences and (ii) $V_{HH}$-library held-out sequences (see Methods). To increase confidence that sequences in these sets can in principle form an NCDB, we restricted the analysis to sequences containing four or five cysteines, including a cysteine at AHo position 40 or 57, which are the dominant cysteine positions observed in four-/five-cysteine nanobody structures (Figure 1). Because Ibex’s training data overlap with our curated NbForge test set, we did not include Ibex in the test-set benchmark; however, Ibex provides a useful comparator for the library-scale NCDB screen where the analysed sequences are distinct from both training and test sets. Under this protocol, NbForge recovered NCDB conformations far more frequently than the other models across both datasets, forming NCDBs in 76.4% (self-distillation-excluded) and 78.4% ($V_{HH}$-library held-out) of sequences, compared with 34.8%/44.1% for NBB2 and 34.1%/39.4% for Ibex (Figure 4B). While NbForge significantly improves in the HCDR3-NCDB recovery of these sequences relative to the other lightweight models, which we show corresponds to lower HCDR3-RMSD, these lightweight predictors are far from achieving complete NCDB recovery. We also note that sequence-based filtering cannot guarantee that every multi-cysteine nanobody sequence from camelid $V_{HH}$ repertoires will in fact adopt a NCDB architecture, and we optimize for this by selecting the most likely of cysteine-pairing partners. Yet, higher NCDB recovery at screening scale implies fewer false negatives when prioritizing candidates with constrained HCDR3 architectures, thereby expanding the set of sequences that can be advanced for experimental validation rather than being discarded due to modelling failures in disulphide-constrained loops.

Beyond improving backbone structure accuracy, blueprint, and NCDB recovery, NbForge yields tangible gains in downstream nanobody-antigen modelling. Using AlphaFold 2.3 Multimer on the NbForge test-set of bound complexes, which unlike AF3 is an open-source model, we compared runs using (i) no nanobody-side evolutionary/template information, (ii) $V_{HH}$ MSA-only, (iii) NBB2 monomer templates, (iv) NbForge monomer templates, and (v) the experimental monomer from the bound complex as a positive-control template (Figure 4C–D). NbForge templating increased the fraction of successful complex predictions (DockQ 0.23) to ~ 47%, compared with ~ 30% for $V_{HH}$ MSA-only or NBB2 templating and ~ 10% with no nanobody-template input (Figure 4D). In addition, NbForge shifted the DockQ distribution toward higher-quality predictions, increasing the proportion of high-accuracy models (DockQ > 0.8) relative to MSA-only/NBB2 and approaching the ceiling set by the experimental-template control (Figure 4D). These gains are achieved without increasing AF2M wall-clock time relative to non-MSA baselines; the main runtime penalty arises from $V_{HH}$ MSA generation in the MSA-only condition (Figure 4C). Finally, several NCDB-bearing nanobodies (purple points) that fail under MSA-only inputs are rescued by NbForge templating, indicating that enforcing nanobody-specific inductive biases can improve complex prediction even for disulphide-constrained HCDR3 loops (Figure 4D). Together, these results suggest that explicitly enforcing nanobody-feature inductive biases during training can translate directly into more reliable complex prediction, strengthening the utility of NbForge as a scalable model for discovery pipelines that prioritize rapid screening, epitope mapping, and structure-guided optimization.

## 3 Discussion

Accurate structural modelling of nanobodies ($V_{HH}s$) is becoming increasingly valuable for nanobody discovery and engineering efforts. However, the regions that typically determine binding and developability potential are also those that remain more difficult to model. HCDR3 loops span broad conformational diversity, and many nanobodies contain additional cysteines that form non-canonical disulphide bridges (NCDBs) that impose covalent constraints on loop geometry. Standard benchmarking of structure-prediction tools focuses on continuous coordinate errors (typically RMSD), yet two models can have similar RMSD while differing in discrete structural commitments that are directly relevant to paratope architecture – most notably the global HCDR3 blueprint (kinked versus extended) and the disulphide connectivity. Taken together, our results support reporting HCDR3-blueprint and disulphide-connectivity recovery alongside coordinate accuracy when assessing nanobody folding models, and demonstrate that incorporating these features as inductive biases improves $V_{HH}$ structure prediction, particularly for long, cysteine-rich HCDR3 loops.

This work introduces two components that make these discrete structural commitments explicit. NbFrame provides a sequence-level prior over kinked and extended HCDR3 regimes using hallmarks concentrated at the CDR3–framework interface, allowing users to flag predictions whose loop placement is inconsistent with the expected blueprint [49, 50, 31]. Practically, such hallmarks also provide a compact set of positions to preserve when proposing framework mutations for humanisation or developability engineering, because altering them can shift the loop into a different blueprint state even when the CDR sequences are unchanged. NbForge encodes these commitments both at the objective level, via an explicit loss term encouraging physically valid disulphide geometry, and at the data level, via self-distillation on a training set filtered to retain only structures that satisfy disulphide bridge formation and the NbFrame blueprint prior. Together, these signals improve HCDR3 RMSD and increase HCDR3 blueprint and NCDB recovery relative to NBB2, while reaching accuracy at par with much deeper models such as AF3 and Boltz (Table 3, 4, Figure 3).

Discovery and engineering pipelines increasingly require both accuracy and throughput. Lightweight predictors such as NBB2 have already been deployed in functional optimisation and developability-focused studies of $V_{HH}s$ [51, 52, 53, 54]. NbForge was designed to deliver backbone accuracy and feature recovery without the latency of large, MSA-dependent deep architectures, enabling repertoire-scale inference where structure prediction becomes a routine filtering step (Figure 4D). Using NbForge monomers as templates for AlphaFold-Multimer increases the fraction of successful complexes (DockQ ≥ 0.23) and shifts predictions toward higher-quality outcomes (DockQ ≥ 0.8) relative to NBB2 templates or the default implementation (Figure 4B, D). Given that AlphaFold-Multimer has been central to multiple design campaigns that yielded successful nanobody designs [55, 51], these results support the practical importance of reliable monomer geometry for downstream complex prediction.

High-quality coordinate and surface representations are also important for residue-burial analysis, and surface-level engineering tasks that are sensitive to loop placement and covalent topology [51, 52, 46, 56], including nanobody humanization via framework resurfacing [52] and structure-guided affinity maturation [57]. Moreover, Bashour *et al*. showed that developability metrics derived from predicted antibody structures are captured more reliably when dynamics are incorporated via molecular dynamics simulations, particularly for properties driven by surface exposure, charge redistribution, and loop flexibility [21]. In this context, backbone accuracy and feature recovery are essential, as simulations initiated from structures with incorrect covalent topology or unrealistic heavy-atom geometries (most notably the cysteine side-chain geometry required for disulphide formation, and the short-range heavy-atom contacts that define the blueprint states) [35] fail to explore physically meaningful conformational sub-states, resulting in misleading flexibility and developability assessments.

NCDBs are common in camelid repertoires and often act as stabilizing topological constraints, while also introducing additional folding and redox sensitivities [58]. Consistent with this dual role, mutagenesis and structural studies show that removing NCDBs can reduce thermal stability and shift reversible unfolding toward aggregation-prone behaviour, and in some cases reduce binding affinity [34, 59]. In *de novo $V_{HH}$* design, introducing additional cysteines into CDRs is typically avoided to limit combinatorial complexity and manage developability risk associated with redox sensitivity. Our results suggest that, with improved disulphide-connectivity recovery, this design constraint can be relaxed when the goal is to explore disulphide-stabilized paratopes. NbForge allows modelling of NCDB-bearing HCDR3s, providing an explicit mechanism to pre-organize paratopes to achieve binding modes inaccessible without NCDBs, and to reduce conformational heterogeneity, likely limiting unfavourable entropy costs upon binding [60]. Moreover, the non-canonical cysteine-pair positional information (Figure 1D, S7) provides concrete guidance for selecting sequence sites when engineering disulphide-stabilized loops in design campaigns.

While accurate monomeric $V_{HH}$ structure prediction and feature recovery helps produce backbones closer to experimentally observed structures, proteins populate a dynamic equilibrium of different interconverting conformational states in solution [60]. Indeed, recent works have focused on developing and using deep-learning methods to predict protein thermodynamic ensembles from sequence [61, 62, 63]. Our results suggest that such predicted ensembles should be benchmarked beyond coordinate uncertainty and dispersion alone, where dispersion can be high either because the model samples alternative but topologically consistent states, or because it produces chemically invalid conformers. For example, ensemble-level metrics such as the fraction of samples satisfying disulphide constraints and the distribution of NbFrame blueprint scores may help distinguish physically plausible heterogeneity from sampling artifacts, and better support downstream interpretations and simulations.

Finally, the ground truth experimental structures in the PDB are a crystallographic or Cryo-EM snapshot, most of which captured in the antigen-bound state [64]. Because our held-out benchmark consists of $V_{HH}s$ taken from bound complexes, our performance metrics quantify agreement with bound conformations rather than the unbound solution ensemble. Antigen binding may shift CDR conformational populations (most prominently HCDR3) consistent with conformational selection, and in some cases additional induced-fit rearrangements are observed [65, 66]. However, a recent analysis of bound and unbound antibody PDB structures suggests that this behaviour is rare, as these two conformations are typically very similar [67]. In general, a predictor that accurately represents the unbound ensemble may be penalized when scored against a bound reference, while a predictor biased toward bound-like geometries could appear to dock better despite being less representative of the $V_{HH}$ free state in solution. This disparity motivates future investigations on paired bound/unbound structures and ensemble-aware reference representations (e.g., MD-derived distributions), to ultimately provide more reliable foundations for downstream dynamics-based developability and functional analyses [60, 21].

We release NbForge and NbFrame as open-source, downloadable software and we make available a user-friendly web server for NbForge structural modelling. We intend these as community resources for routine, high-throughput nanobody modelling, where rapid access to reliable unbound structures enables earlier and broader use of structure-informed decision making.

## 4 Methods

### 4.1 NbFrame: HCDR3 conformation classification

#### Structural database curation for NbFrame training

A total of 1,056 $V_{HH}$ structures were initially retrieved from SAbDab databse [37]. Structures were superimposed to a reference nanobody (PDB: 2P49, chain B) using framework residues only and a quality filter excluded structures with framework RMSD > 2.0 Å to the reference, removing 51 poorly resolved or domain-swapped structures and yielding 1,005 structures. To prevent data leakage from redundant sequences, we performed CDR-based deduplication. Structures sharing identical HCDR1+HCDR2+HCDR3 sequences (concatenated as a single string) were identified, and one representative per unique combination was retained, yielding a final dataset of 929 unique $V_{HH}$ structures. The representative structure was chosen based on minimal number of missing residues (i.e. without assigned coordinates) in the core region (i.e. excluding 4 residues at N- and C-terminus) and highest resolutions. Sequences were aligned and numbered according to the AHo numbering scheme [68], providing a consistent 149-position alignment across structures. HCDR boundaries were defined as: HCDR1 (AHo 27–42), CDR2 (AHo 57–69), and HCDR3 (AHo 108–138).

A temporal split based on PDB accession codes was used for NbFrame only, where PDB structures with ID’s beginning with “9” were designated as the held-out NbFrame test set (*n* = 100) (Figure S10), while remaining structures formed the NbFrame training set.

#### NbFrame Structural feature calculation

Six features were calculated for each structure to characterize HCDR3 conformation:

##### Backbone angles.

The α (virtual bond angle) and τ (virtual torsion angle) were calculated at both the N-terminal and C-terminal stems of the HCDR3 loop. For the N-terminal stem, angles were measured at AHo positions 105–108; for the C-terminal stem, at positions 135–138. Virtual bond angles were defined as:

(1) $$\alpha =arccos\left(\frac{{\vec{v}}_{1}\cdot {\vec{v}}_{2}}{\left|{\vec{v}}_{1}\right|\left|{\vec{v}}_{2}\right|}\right)$$

where ${\vec{v}}_{1}$ and ${\vec{v}}_{2}$ are vectors between consecutive Cα atoms.

##### Contact density.

Atomic contacts between HCDR3 residues and FR2 residues (AHo positions 44–55) were counted using a distance cut-off of 4.5 Å. CDR3 stem residues (AHo positions 108, 109, 136, 137, 138) were excluded from contact counting. Contact density was normalized by the full CDR3 length to provide a length-independent measure:

(2) $$\mathrm{contact}\;\mathrm{density}=\frac{N_{\mathrm{contacts,}\;\mathrm{non-stem}}}{{\mathrm{Length}}_{CDR3}}$$

where $N_{\mathrm{contacts,}\;\mathrm{non-stem}}$ is the number of contacts between non-stem CDR3 residues and FR2, and ${Length}_{\mathrm{CDR3}}$ is the full CDR3 length.

##### FR2 relative solvent accessibility.

Solvent accessible surface area (SASA) was calculated using FreeSASA [69] with default parameters (probe radius 1.4 Å). Relative solvent accessible surface area (RSASA) was computed for key FR2 positions (44 and 54) by normalizing with theoretical maximum SASA values derived from exhaustive conformational sampling of Gly-X-Gly tripeptides [70]. The feature fr2_rsa_key was defined as the mean RSA of AHo positions 44 and 54.

#### Unsupervised clustering and manual labelling of kinked and extended blueprints

To identify natural groupings in the six-dimensional feature space, we applied Gaussian Mixture Model (GMM) clustering with full covariance matrices using scikit-learn [71]. Features were standardized using StandardScaler prior to clustering. Model selection based on silhouette score indicated an optimal two-cluster solution (silhouette = 0.34), consistent with the hypothesized kinked/extended dichotomy.

For ground truth establishment, we sampled 50 structures from each GMM cluster, including: i) structures from each cluster core (GMM probability > 0.99), (ii) structures from near-boundary regions (probability 0.90–0.99), and (iii) structures from the true boundary (probability 0.10–0.90). Each structure was manually labelled as Kinked (K) or Extended (E) based on visual assessment of HCDR3 conformation. Labelling criteria were kinked: HCDR3 bends back toward the framework and makes contacts with FR2 residues; Extended: HCDR3 projects outward with minimal or no FR2 contact.

Manual classification yielded 60 extended and 40 kinked labels from the 100 sampled structures, reflecting 80% concordance between GMM clustering and manual labels. Analysis of the 20 discordant cases revealed that GMM clustering primarily captured C-terminal backbone geometry (particularly $\alpha_{C}$ angles), whereas the structural-biology definition of kinked conformations requires not only kinked-like angles but also physical CDR3-FR2 contacts that shield hydrophobic framework residues from solvent. Among the discordant structures, 15 exhibited kinked-like angles but lacked FR2 contacts (contact density = 0) and were therefore classified as extended, while only 5 structures from the extended cluster were reclassified as kinked upon visual inspection.

#### NbFrame structure classifier training

A logistic regression classifier was trained on the 100 manually labelled structures, using stratified 5-fold cross-validation. Features were standardized using StandardScaler, and the scaler and classifier were combined into a single pipeline. Model performance was evaluated using cross-validation on the training set (ROC-AUC = 0.96) and then on the 100 held-out test structures with manual labels (ROC-AUC = 0.99, accuracy = 94%).

The NbFrame structural classifier computes the probability of a kinked conformation as:

(3) $$P(\mathrm{kinked})=\sigma \left(\sum_{i=1}^{6}\;w_{i}\cdot \frac{x_{i}\;-\;\mu_{i}}{\sigma_{i}}+b\right)$$

where $\sigma (z)=1/\left(1+e^{-z}\right)$ is the sigmoid function, $x_{i}$ is the raw feature value, $\mu_{i}$ and $\sigma_{i}$ are the per-feature mean and standard deviation from the training set (used for standardization), $w_{i}$ is the learned coefficient for feature i, and b=−0.352 is the intercept term. The complete parametrisation is provided in Table 6.

For deployment, confidence thresholds were implemented: predictions with P(kinked) > 0.7 were labelled as “kinked,” P(kinked) < 0.3 as “extended,” and intermediate probabilities as “unclear.” The trained classifier was applied to all 929 structures, yielding 480 Kinked (51.7%), 356 Extended (38.3%), and 93 Unclear (10.0%) predictions.

#### NbFrame sequence classifier

To enable sequence-based predictions, we developed a logistic regression classifier based on framework sequence features associated with HCDR3 blueprint. For each (AHo position, amino acid) combination in framework regions (in AHo numbering - FR1: positions 1–26; FR2: positions 43–56; FR3: positions 70–107; FR4: positions 139–149), we computed the ${log}_{2}$ fold-change of frequencies $\left({Log}_{2}FC\right)$ between kinked and extended structures:

(4) $${Log}_{2}FC(p,a)={log}_{2}\left(\frac{f_{K}(p,a)\;+\;\epsilon }{f_{E}(p,a)\;+\;\epsilon }\right)$$

where $f_{K}(p,a)$ and $f_{E}(p,a)$ are the frequencies of amino acid a at AHo position p in Kinked and Extended structures, respectively, and ϵ=0.5/N is a pseudo-count to avoid division by zero (N is the number of training structures).

For downstream analysis, we retained only those (p,a) combinations that satisfied: (i) Fisher’s exact test *p* < 0.05, and (ii) total observations ≥ 30 (across Kinked and Extended classes). For each combination, we computed: (i) point-biserial correlation with binary class labels in the training set structures (discriminative power), and (ii) Pearson correlation with structure classifier probabilities of training-set structures (consistency with structural predictions). The combined score was defined as the product of absolute correlations $\left(\left|r_{\mathrm{label}}\right|\times \left|r_{\mathrm{struct}}\right|\right)$, and the top 20 candidates by this metric were selected as hallmarks, because using 20 features maximised the E/K discrimination performance on the training set. This approach prioritizes features that are both discriminative and aligned with the structure classifier, such that the sequence classifier approximates structure-based classification from sequence alone. The resulting hallmarks are concentrated at biologically relevant positions: FR1 (positions 12, 15, 17), FR2 (positions 44, 51, 54, 56), and FR3 (positions 85, 103, 107). Features from CDR regions were excluded to avoid potential bias from CDR sequences in engineered or synthetic $V_{HH}s$.

A logistic regression model was trained on 829 sequences with known structures using soft labels from the structure classifier (P(kinked)) treated as continuous probability. For a given AHo-aligned sequence S, features are encoded as follows: for each hallmark j∈{1,…,20}, defined by position $p_{j}$ and amino acid $a_{j}$ with associated ${Log}_{2}{FC}_{j}$ value (Table 7), the feature $x_{j}$ is set to ${Log}_{2}{FC}_{j}$ if the sequence has amino acid $a_{j}$ at position $p_{j}$, and 0 otherwise:

(5) $$x_{j}=\left\{\begin{array}{ll} Log2{FC}_{j} & \;\mathrm{if}\;S\left[p_{j}\right]=a_{j}\\ 0 & \;\mathrm{otherwise}\; \end{array}\right.$$

The probability of a kinked conformation is then computed as:

(6) $$P(\mathrm{kinked})=\sigma \left(\sum_{j=1}^{20}\;w_{j}\cdot \frac{x_{j}\;-\;\mu_{j}}{\sigma_{j}}+b\right)$$

where $\sigma (z)=1/\left(1+e^{-z}\right)$ is the sigmoid function, $\mu_{j}$ and $\sigma_{j}$ the mean and standard deviation of feature $x_{j}$ computed from the training set, and $w_{j}$ and b are the learned model coefficients and bias, respectively.

The model was evaluated on the 100 held-out structures with manual labels as ground truth, achieving ROC-AUC of 0.94 and accuracy of 86%.

#### NbFrame software implementation

NbFrame is implemented in Python and is available as an open-source package on GitHub (https://github.com/Mateusz-Jaskolowski/NbFrame). The structure classifier accepts PDB or mmCIF files and outputs predicted class and probability. The sequence classifier accepts amino acid sequences (with optional automatic AHo alignment) and outputs probability of kinked or extended.

### 4.2 NbForge Model Architecture

#### Disulphide bridge loss

We adapt the architecture and base training objectives of ABody-Builder3 [72, 73], which in turn are built on the structure module of AlphaFold2. We augment these with a composite disulphide-bridge loss to encourage physically realistic cysteine pairing and geometry based on ground-truth disulphide bridges of $V_{HH}s$. All disulphide-specific loss terms are applied only to cysteine residues and are annealed during training.

Distance-based disulphide terms use linear hinge penalties; the S−S−Cβ bond angle is regularized with a Huber loss, while the $\chi_{3}$ dihedral is supervised using a bimodal wrapped likelihood.

For a nanobody containing N∈{2,3,4,5} cysteine residues, let $s_{i}\in R^{3}$ and $c_{i}\in R^{3}$ denote the predicted SG and Cβ atom coordinates of cysteine i, respectively, and let $s_{i}^{gt}$ denote the corresponding ground-truth SG coordinate. All distances are measured in Å.

#### Cysteine pairing

To identify disulphide bonds, we compute the ground-truth SG–SG distances

(7) $$d_{ij}^{gt}={\left\|s_{i}^{gt}-s_{j}^{gt}\right\|}_{2}$$

for all cysteine pairs using i<j to prevent double counting. Pairs satisfying

(8) $$1.42\leq d_{ij}^{gt}\leq 3.00$$

are considered candidates based on the distribution of SG-SG distances in ground-truth structures. From these, we greedily select a non-overlapping set of bonded cysteine pairs ℬ, such that each cysteine participates in at most one bond. All remaining cysteine pairs form the complementary set ${ℬ}^{\prime }$.

#### SG–SG distance band and non-bonded repulsion

Let

(9) $$d_{ij}={\left\|s_{i}-s_{j}\right\|}_{2}$$

denote the predicted SG–SG distance. We apply a hinge-style penalty that constrains bonded cysteine pairs to a narrow distance band [1.84, 2.26] Å and repels additional cysteines to prevent unphysical “clusters” of cysteine residues “attracting” each others (by pushing any upaired cysteine to have their distance at least 4.1 Å away from the paired cysteines):

(10) $${ℒ}_{SG}=\sum_{(i,j)\in ℬ}\left[max\left(d_{ij}-2.26,0\right)+max\left(1.84-d_{ij},0\right)\right]+\sum_{(i,j)\in {ℬ}^{\prime }}max\left(4.1-d_{ij},0\right).$$

#### Cβ−Cβ distance prior

For bonded cysteine pairs, we regularize the predicted Cβ−Cβ distance using a flat-bottom hinge loss that penalizes deviations outside a target interval [2.85, 4.50] Å

(11) $$r_{ij}={\left\|c_{i}-c_{j}\right\|}_{2}$$

using a flat-bottom hinge loss:

(12) $${ℒ}_{CB}=\sum_{(i,j)\in ℬ}\left[max\left(r_{ij}-4.50,0\right)+max\left(2.85-r_{ij},0\right)\right].$$

#### SG–SG–C β bond angle prior

For each bonded cysteine pair (i,j), we compute the two S−S−Cβ angles

(13) $$\theta_{i}=∠\left(c_{i}-s_{i},s_{j}-s_{i}\right),$$

(14) $$\theta_{j}=∠\left(s_{i}-s_{j},c_{j}-s_{j}\right),$$

and penalize deviations from a target angle θ=2.0 rad using a Huber loss with transition parameter δ=0.04:

(15) $${ℒ}_{\mathrm{ang}\;}=\sum_{(i,j)\in ℬ}\left[{\mathrm{Huber}}_{0.04}\left(\theta_{i}-\theta \right)+{\mathrm{Huber}}_{0.04}\left(\theta_{j}-\theta \right)\right].$$

### disulphide $\chi_{3}$ dihedral prior

For each bonded cysteine pair, we define the disulphide $\chi_{3}$ dihedral angle

(16) $$\phi_{ij}=dihedral\left(c_{i},s_{i},s_{j},c_{j}\right).$$

We impose a bimodal wrapped Gaussian penalty corresponding to the two canonical disulphide rotamers, providing a smooth objective that avoids hard mode assignment:

(17) $${ℒ}_{\chi_{3}}=\sum_{(i,j)\in ℬ}-\log\left(\exp\left[-\frac{1}{2}\frac{\Delta {\left(\phi_{ij},1.6601\right)}^{2}}{0.50^{2}}\right]+\exp\left[-\frac{1}{2}\frac{\Delta {\left(\phi_{ij},-1.7951\right)}^{2}}{0.50^{2}}\right]\right),$$

where Δ(⋅,⋅) denotes the angular difference wrapped to (−π,π].

### disulphide distogram loss

In addition to coordinate-based penalties, we supervise the predicted residue–residue distogram for bonded cysteine pairs only. Let $\ell_{p_{i}p_{j}}\in R^{64}$ denote the predicted distogram logits between the sequence positions $p_{i}$ and $p_{j}$. We define a target bin corresponding to a SG–SG distance of 2.05 Å and apply a symmetric cross-entropy loss:

(18) $${ℒ}_{\mathrm{disto}}=\sum_{(i,j)\in ℬ}\left[CE(\ell_{p_{i}p_{j}},b)+CE(\ell_{p_{j}p_{i}},b)\right].$$

### Annealing

The total disulphide objective is given by

(19) $${ℒ}_{\mathrm{disulphide}}=0.5{ℒ}_{SG}+0.25{ℒ}_{CB}+10^{-4}\left({ℒ}_{ang}+{ℒ}_{\chi_{3}}\right)+1\;{ℒ}_{\mathrm{disto}}.$$

and is multiplied by an annealing factor that increases linearly from 0 to 1 over the first 100000 training steps.

### Training and inference

A first version of the model was trained only on the $V_{H}/V_{HH}$ experimental structures for 500,000 steps. This model was used to create the self-distillation set (Figure 2). Then, another version of the model was trained only on the filtered self-distillation dataset always for 500,000 steps. This model was then fine-tuned on the experimental $V_{H}/V_{HH}$ structures for 500,000 steps. When performing inference, unless stated otherwise, structures predicted by NbForge were subjected to restrained energy minimisation in OpenMM using the AMBER14SB protein force field, as done by NBB2 [45, 74, 75], including the application of harmonic positional restraints to N,Cα,C and Cβ atoms to keep the refined backbone close to its original prediction.

### 4.3 Evaluation of blueprint and NCDB features

#### Blueprint recovery

The NbFrame structure classifier was used to evaluate all ground truth, experimental structures of the test set. The classifier threshold of less than 0.25 is used to classify HCDR3 loop to be extended, in between 0.25 and 0.55 are deemed to be unclear, and greater than 0.55 is classified as kinked, as per the accuracy analysis performed in Figure S11. We then evaluated the predicted structures from each model using the same NbFrame structure classifier, and the overlap into the three different bins is used to measure the accuracy of each models’ structure relative to the experimental structures (Figure 4B, Table 3).

#### NCDB recovery

Both canonical and non-canonical disulphide bridges from all the SAbDab experimental $V_{HH}s$ are evaluated. Only SG-SG distances of less than 3 Å were kept for evaluation based on previous work that used a similar distance threshold [76]. From this set of disulphide bridges, the 99th percentile of SG-SG distances was used (2.46 Å) as a distance threshold cutoff for the evaluation. After prediction, the expected disulphide-bridges and their respective cysteine SG atom distances were calculated and if they satisfied the 2.46 Å threshold they were deemed to have formed the disulphide bridge. The violations of this 2.46 Å SG-SG distance threshold for each predicted structures are reported in Table 3.

### 4.4 NCDB recovery in large sequence datasets

Two large sequence datasets were folded with the three lightweight models (NbForge, NBB2, and Ibex) and evaluated for their NCDB recovery (Figure 4). Dataset 1 (self distillation-excluded sequences) was derived from an initial set of 154,115 sequences for which structures were predicted with the teacher $V_{H}/V_{HH}$ model. After filtering out structures for structural violations (Figure 2D, see Results), 95,540 sequences remained. From these, we retained only sequences with (i) 4–5 cysteines total across the full $V_{HH}$ domain sequence, (ii) a cysteine at AHo position 40 and/or 57 (dominant cysteine position shown in Figure 1B), and (iii) at least one cysteine in HCDR3 (i.e., enabling a putative 40/57–HCDR3 NCDB), yielding 31,172 sequences (SD-excluded dataset). Dataset 2 (held-out $V_{HH}$ library) started from sequences retained after the “cluster by 80% MMSEQ” and “remove singletons” steps (Figure 2D). We removed any sequences overlapping Dataset 1 (exact sequence identity) and then applied the same cysteine-based criteria as done for Dataset 1, yielding 132,103 sequences.

### 4.5 AlphaFold 2.3 Multimer $V_{HH}$-antigen complex prediction

All nanobodies from the NbForge test-set are in the bound state against their respective antigen in their PDBs. Multi sequence alignment (MSAs) were generated separately for both the antigen and the nanobody against the UniRef^30^ clustered database and the ColabFold environmental sequence database using MMseqs2 [42]. Structure prediction was performed using ColabFold running AlphaFold 2.3 Multimer (AF2M) [13, 77, 78], with custom monomeric $V_{HH}$ templates either from experimental structures, NBB2, or NbForge modelled structures, with the precomputed antigen MSAs from above supplied as input. Predictions used the default five model-prediction [78] with three recycle iterations and a single random seed (0). DockQ value was calculated on the whole nanobody-antigen complex, and the top DockQ score was selected from the 5 replicates of each complex prediction respectively [79].

### 4.6 Heavy-model monomer inference pipelines

AlphaFold3 (AF3) structure prediction was performed in single-chain (monomer) mode for all $V_{HH}$ sequences analysed in this work. For each sequence, a multiple sequence alignment (MSA) was generated using a local MMseqs2-based ColabFold-style pipeline [77], producing an .a3m alignment that was provided to AF3 as a custom MSA input. Structural templates were not provided manually; instead, AF3 was run with template usage enabled and performed automatic template selection via its internal template search. AF3 inference was run using a single model seed; this seed produces multiple diffusion samples (five by default), from which the highest-ranked model under AF3’s default ranking/scoring scheme was retained as the single structural prediction for each sequence. This top-ranked model was used for all downstream analyses and visualisation.

Boltz1 structures were predicted with the official Boltz implementation using the Boltz-1 model weights. Each target sequence was provided as a single-chain input ($V_{HH}$ domain only), and we generated one predicted structure per target (one diffusion sample) for downstream evaluation, consistent with the default (-diffusion_samples 1). Inference used the default Boltz prediction hyperparameters: 3 recycling steps (-recycling_steps 3) and 200 sampling steps (-sampling_steps 200). Multiple sequence alignments (MSAs) were used for the primary Boltz1 predictions. MSAs were generated using the standard Boltz workflow via the MMseqs2 MSA server (-use_msa_server), which queries the ColabFold MMseqs2 API endpoint by default. For monomeric inputs, a single .a3m MSA is auto-generated when -use_msa_server is enabled. No structural templates were provided for Boltz1 predictions, which (unlike AlphaFold3) cannot use input templates in its inference pipeline.

## Supplementary Material

Supplement 1

## Figures and Tables

**Table 1:** NbFrame classifier performance on held-out test structures (*n* = 100).

Classifier	ROC-AUC	PR-AUC	Accuracy	Features

Structure-based	0.994	0.995	94.0%	6
Sequence-based	0.939	0.956	86.0%	20

**Table 2:** Summary statistics of $V_{HH}$ datasets used in this study, including mean HCDR3 length, frequency of non-canonical disulphide bonds (NCDB), and frequency of kinked HCDR3 conformations.

Dataset	Size (N)	HCDR3 length	NCDB(%)	Kinked (%)

SAbDab (all $V_{HH}$ chains)	3124	14.7 ± 4.5	17.0	69.6
SAbDab (unique $V_{HH}$ sequences)	892	14.4 ± 4.3	13.9	68.6
$V_{HH}$ Library	2.22 × 10^7^	15.2 ± 4.5	26.3	61.8
NanobodyBuilder2 test set	30	14.0 ± 4.5	2.5	80.0
NbForge test set	47	17.8 ± 4.3	31.3	62.5

Note: the kinked and extended evaluations are performed using the NbFrame sequence and structure classifier for the library dataset and PDBs, respectively, and the NCDBs from the library sequences are assumed to form in all sequences with 4 and 5 cysteines.

**Table 3:** Coordinate accuracy and feature recovery on the NbForge test set. Backbone (CA, N, CO, O) RMSDs are reported as median with bootstrap 95% confidence intervals (CI) in Å (*n* = 47) after framework-region superimposition. Confidence intervals were estimated using non-parametric bootstrap resampling with 10,000 resamples. Kinked/extended (K/E) correctness is computed over all test structures, while non-canonical disulphide bond (NCDB) recovery is computed over the subset containing a non-canonical disulphide bond (*n* = 15), see Methods.

Metric	AF3	Boltz1	NBB2	NbForge

All	1.39 [1.05, 1.53]	1.52 [1.23, 1.74]	1.62 [1.51, 1.94]	1.47 [1.37, 1.63]
FR	0.70 [0.57, 0.72]	0.65 [0.59, 0.73]	0.79 [0.67, 0.85]	0.72 [0.68, 0.75]
HCDR1	1.19 [1.07, 1.53]	1.28 [1.02, 1.63]	1.72 [1.53, 2.17]	1.65 [1.34, 1.78]
HCDR2	0.80 [0.67, 1.25]	0.89 [0.63, 1.33]	1.17 [0.89, 1.44]	1.18 [0.98, 1.48]
HCDR3	2.95 [2.16, 3.66]	3.39 [2.51, 3.74]	3.45 [2.99, 4.85]	2.95 [2.77, 3.65]

K/E recovery (%)	93.6	89.4	80.9	91.5
NCDB recovery (%)	93.3	73.0	26.7	86.7

**Table 4:** Median Backbone coordinate accuracy (RMSD, Å) stratified by presence of a non-canonical disulphide bond (NCDB *n* = 15) or by its absence (no NCDB *n* = 32). RMSDs are calculated for all backbone heavy atoms following framework superimposition and reported as median with bootstrap 95% confidence intervals.

NCDB	Region	AF3	Boltz1	NBB2	NbForge

no NCDB	FR	0.70 [0.58, 0.73]	0.66 [0.58, 0.74]	0.76 [0.62, 0.87]	0.73 [0.68, 0.83]
	HCDR1	1.20 [0.94, 1.67]	1.18 [0.96, 1.55]	1.74 [1.31, 2.23]	1.61 [1.15, 2.11]
	HCDR2	0.68 [0.53, 1.06]	0.74 [0.58, 1.42]	0.95 [0.83, 1.43]	1.05 [0.89, 1.33]
	HCDR3	2.75 [1.59, 3.55]	2.92 [2.11, 4.12]	3.56 [2.91, 5.01]	2.95 [2.51, 3.71]

NCDB	FR	0.68 [0.52, 0.76]	0.65 [0.54, 0.77]	0.85 [0.67, 0.88]	0.70 [0.64, 0.85]
	HCDR1	1.19 [0.85, 1.97]	1.63 [0.83, 2.73]	1.72 [1.48, 2.62]	1.65 [1.39, 1.90]
	HCDR2	1.26 [0.70, 1.37]	1.26 [0.63, 1.79]	1.44 [1.08, 1.73]	1.26 [1.00, 1.64]
	HCDR3	2.97 [2.12, 3.70]	3.58 [2.30, 3.74]	3.45 [2.99, 4.53]	2.95 [2.69, 3.53]

**Table 5:** Wall-clock inference timings on the NbForge test-set (*n* = 47) are reported in seconds and averaged over three independent runs on an NVIDIA Quadro RTX 8000 GPU and an Intel Xeon Platinum 8263.

Metric	AF3	Boltz1	NBB2	NbForge-GPU	NbForge-CPU

Total time (s; 3 runs)^†‡^	2676.9 (1841.8)	2961.4 (2088.1)	410	6.1 (72.2)	10.0 (128.1)
Per-structure time (s)^†‡^	57.0 (39.2)	63.0 (44.4)	4.5	0.13 (1.54)	0.21 (2.73)

† For AlphaFold3 and Boltz1, values in parentheses correspond to predictions performed without MSA/template search.

‡ For NbForge, values in parentheses correspond to the extra time taken by the optional OpenMM relaxation step, with monomer inference times preceding. NBB2 timings are CPU-only.

**Table 6:** NbFrame structure classifier parameters. Features are standardized by subtracting the mean (μ) and dividing by the standard deviation (σ) before applying the logistic regression coefficients (w). Training set: *n* = 100 manually labelled structures.

Feature	Mean (μ)	Std (σ)	Coefficient (w)

$\alpha_{N}$ (degrees)	−62.09	133.53	+0.11
$\tau_{N}$ (degrees)	126.22	11.47	+0.60
$\alpha_{C}$ (degrees)	−13.31	101.63	+0.48
$\tau_{C}$ (degrees)	109.68	13.78	−0.42
contact density	0.29	0.31	+1.86
fr2_rsa_key	0.14	0.09	−1.16

Intercept: *b* = −0.352

**Table 7:** Sequence hallmark features used for the NbFrame sequence classifier, derived from 829 unique $V_{HH}$ sequences from SAbDab [37], with blueprint labels assigned by the structure classifier. Features represent specific (AHo position, amino acid) combinations with corresponding enrichment in Kinked versus Extended structures. Log_2_FC indicates Fold-Change of frequencies; positive values indicate enrichment in Kinked structures. Frequencies are provided as percentages from a total of 478 kinked and 351 extended structures, and the p-values is from a Fisher’s exact test.

Region	AHo Position	AA	Log_2_FC	*p*-value	% Kinked	% Extended

*Framework 1*						
FR1	12	S	+1.14	5.4 × 10^−5^	17.6	8.0
FR1	12	L	−0.14	2.4 × 10^−3^	78.7	86.9
FR1	15	P	−0.58	3.9 × 10^−6^	32.2	48.1
FR1	15	A	+0.38	3.9 × 10^−5^	62.3	47.9
FR1	17	D	+2.32	8.5 × 10^−5^	7.1	1.4

*Framework 2*						
FR2	44	F	+1.72	< 10^−45^	81.0	24.5
FR2	44	Y	−2.77	< 10^−45^	9.0	61.3
FR2	51	E	+0.72	1.2 × 10^−20^	79.1	47.9
FR2	51	Q	−2.63	1.7 × 10^−25^	5.2	32.5
FR2	54	F	+1.24	2.1 × 10^−15^	45.2	19.1
FR2	54	G	+2.21	9.0 × 10^−18^	29.1	6.3
FR2	54	L	−2.34	1.8 × 10^−26^	7.3	37.0
FR2	54	W	−1.00	6.5 × 10^−6^	11.9	23.9
FR2	56	A	−0.20	1.7 × 10^−3^	68.0	78.1

*Framework 3*						
FR3	85	S	−1.73	3.7 × 10^−7^	4.4	14.5
FR3	85	A	+0.21	5.9 × 10^−5^	84.3	72.6
FR3	103	V	−0.29	5.4 × 10^−7^	68.0	83.2
FR3	103	I	+1.45	1.3 × 10^−4^	11.7	4.3
FR3	107	A	+1.20	< 10^−45^	86.8	37.9
FR3	107	N	−2.46	8.3 × 10^−21^	5.2	28.8

## Data Availability

The NbFrame source code, sequence and structure models and weights are available at https://github.com/Mateusz-Jaskolowski/NbFrame. The NbForge source code and weights are available at https://gitlab.doc.ic.ac.uk/sormanni-lab/nbforge. All the parsed training, validation and testing datasets employed in this study are available alongside the source code.
