# Supplementary material for: Disulphide and sequence-encoded conformational priors guide nanobody structure prediction
Source: bioRxiv. 2026 Feb 13:2026.02.13.705647. Preprint. [Version 1] doi: 10.64898/2026.02.13.705647 (PMC13119344; doi:10.64898/2026.02.13.705647)
Supplement: Supplement 1 [file NIHPP2026.02.13.705647v1-supplement-1.pdf]

bioRxiv preprint doi: https://doi.org/10.64898/2026.02.13.705647; this version posted February 13, 2026. The copyright holder for this preprint (which was not certified by peer review) is the author/funder, who has granted bioRxiv a license to display the preprint in perpetuity. It is made available under aCC-BY 4.0 International license.

9 Supplementary Information

Table S1: RMSD comparisons for NbForge versus baselines on the NbForge test set ($n = 47$). RMSDs are backbone-atom RMSD (Å) after framework superimposition. Δ values are mean paired differences (NbForge minus different baseline models; negative values indicate improvement). P-values are from two-sided paired Wilcoxon signed-rank tests, paired t-tests, and paired sign-flip permutation tests on the median paired difference (20,000 permutations).

		**Δ (NbForge – model RMSD)**			**p-value**								
Region	NbForge mean RMSD	NBB2	AF3	Boltz1	Wilcoxon			*t*-test			Permutation (median)		
					NBB2	AF3	Boltz1	NBB2	AF3	Boltz1	NBB2	AF3	Boltz1
All backbone	1.57	-0.21	0.10	0.01	1.4e-04	0.27	0.95	1.5e-04	0.25	0.88	1.5e-03	0.65	0.65
Framework	0.79	-0.02	0.09	0.10	0.61	<1e-04	<1e-04	0.33	<1e-04	<1e-04	0.76	<1e-04	<1e-04
HCDR1	1.65	-0.22	0.09	-0.01	0.026	0.33	0.77	0.028	0.58	0.97	0.014	0.15	0.44
HCDR2	1.36	0.01	0.30	0.15	0.92	<1e-04	0.048	0.83	1.1e-04	0.056	0.77	1.3e-03	0.042
HCDR3	3.40	-0.64	0.15	0.01	2.7e-04	0.49	0.90	2.7e-04	0.56	0.97	6.0e-04	0.88	0.77

Table S2: RMSD comparisons for AF3 versus baselines on the test set ($n = 47$). RMSDs are backbone-atom RMSD (Å) after framework superimposition. Δ values are mean paired differences (AF3 minus different baseline models; negative values indicate improvement). P-values are from two-sided paired Wilcoxon signed-rank tests, paired t-tests, and paired sign-flip permutation tests on the median paired difference (20,000 permutations).

		**Δ (AF3 – model RMSD)**			**p-value**								
Region	AF3 mean RMSD	NbForge	NBB2	Boltz1	Wilcoxon			*t*-test			Permutation (median)		
					NbForge	NBB2	Boltz1	NbForge	NBB2	Boltz1	NbForge	NBB2	Boltz1
All backbone	1.47	-0.10	-0.30	-0.08	0.27	0.0044	0.094	0.25	0.0023	0.25	0.65	0.014	0.19
Framework	0.69	-0.09	-0.11	0.01	<1e-04	<1e-04	0.47	<1e-04	<1e-04	0.58	<1e-04	<1e-04	0.44
HCDR1	1.57	-0.09	-0.30	-0.09	0.33	0.024	0.14	0.58	0.056	0.48	0.15	0.024	0.27
HCDR2	1.06	-0.30	-0.29	-0.16	<1e-04	<1e-04	0.14	<1e-04	<1e-04	0.026	0.0019	0.0035	0.76
HCDR3	3.25	-0.15	-0.78	-0.14	0.49	0.017	0.21	0.56	0.0076	0.53	0.88	0.066	0.44

Table S3: RMSD comparisons for Boltz1 versus baselines on the test set ($n = 47$). RMSDs are backbone-atom RMSD (Å) after framework superimposition. Δ values are mean paired differences (Boltz1 minus different baseline models; negative values indicate improvement). P-values are from two-sided paired Wilcoxon signed-rank tests, paired t-tests, and paired sign-flip permutation tests on the median paired difference (20,000 permutations).

		**Δ (Boltz1 – model RMSD)**			**p-value**								
Region	Boltz1 mean RMSD	NbForge	NBB2	AF3	Wilcoxon			*t*-test			Permutation (median)		
					NbForge	NBB2	AF3	NbForge	NBB2	AF3	NbForge	NBB2	AF3
All backbone	1.56	-0.01	-0.22	0.08	0.95	0.032	0.094	0.88	0.020	0.25	0.65	0.099	0.19
Framework	0.69	-0.10	-0.12	-0.01	<1e-04	<1e-04	0.47	<1e-04	<1e-04	0.58	<1e-04	<1e-04	0.44
HCDR1	1.66	0.01	-0.21	0.09	0.77	0.031	0.14	0.97	0.13	0.48	0.44	0.025	0.27
HCDR2	1.21	-0.15	-0.13	0.16	0.048	0.039	0.14	0.056	0.058	0.026	0.042	0.065	0.76
HCDR3	3.39	-0.01	-0.65	0.14	0.90	0.030	0.21	0.97	0.029	0.53	0.77	0.097	0.44

Table S4: RMSD comparisons for NBB2 versus baselines on the test set ($n = 47$). RMSDs are backbone-atom RMSD (Å) after framework superimposition. Δ values are mean paired differences (NBB2 minus different baseline models; negative values indicate improvement). P-values are from two-sided paired Wilcoxon signed-rank tests, paired t-tests, and paired sign-flip permutation tests on the median paired difference (20,000 permutations).

		**Δ (NBB2 – model RMSD)**			**p-value**								
Region	NBB2 mean RMSD	NbForge	AF3	Boltz1	Wilcoxon			*t*-test			Permutation (median)		
					NbForge	AF3	Boltz1	NbForge	AF3	Boltz1	NbForge	AF3	Boltz1
All backbone	1.78	0.21	0.30	0.22	1.4e-04	0.0044	0.032	1.5e-04	0.0023	0.020	1.5e-03	0.014	0.097
Framework	0.81	0.02	0.11	0.12	0.61	<1e-04	<1e-04	0.33	<1e-04	<1e-04	0.76	<1e-04	<1e-04
HCDR1	1.87	0.22	0.30	0.21	0.026	0.024	0.031	0.028	0.056	0.13	0.014	0.024	0.026
HCDR2	1.35	-0.01	0.29	0.13	0.92	<1e-04	0.039	0.83	<1e-04	0.058	0.77	0.0035	0.064
HCDR3	4.03	0.64	0.78	0.65	2.7e-04	0.017	0.030	2.7e-04	0.0076	0.029	6.0e-04	0.066	0.10

bioRxiv preprint doi: https://doi.org/10.64898/2026.02.13.705647; this version posted February 13, 2026. The copyright holder for this preprint (which was not certified by peer review) is the author/funder, who has granted bioRxiv a license to display the preprint in perpetuity. It is made available under aCC-BY 4.0 International license.

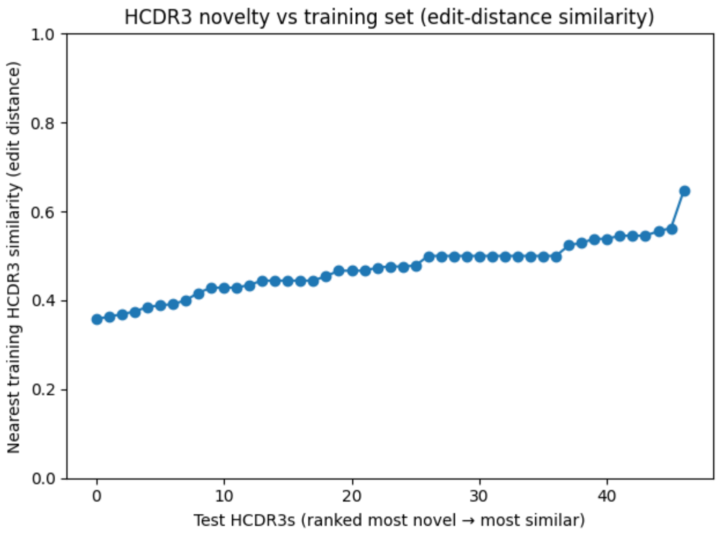

Figure S1: HCDR3 nearest-neighbour similarity to the training and validation set. For each held-out test HCDR3 sequence, the maximum similarity to any training- or validation-set HCDR3 was computed using Levenshtein edit distance and normalized by the maximum HCDR3 length $\left(\text{similarity} = 1 - \frac{d}{\max\left(L_{\text{test}}, L_{\text{train}}\right)}\right)$. Test HCDR3s are ranked from most novel (left) to most similar (right).

bioRxiv preprint doi: https://doi.org/10.64898/2026.02.13.705647; this version posted February 13, 2026. The copyright holder for this preprint (which was not certified by peer review) is the author/funder, who has granted bioRxiv a license to display the preprint in perpetuity. It is made available under aCC-BY 4.0 International license.

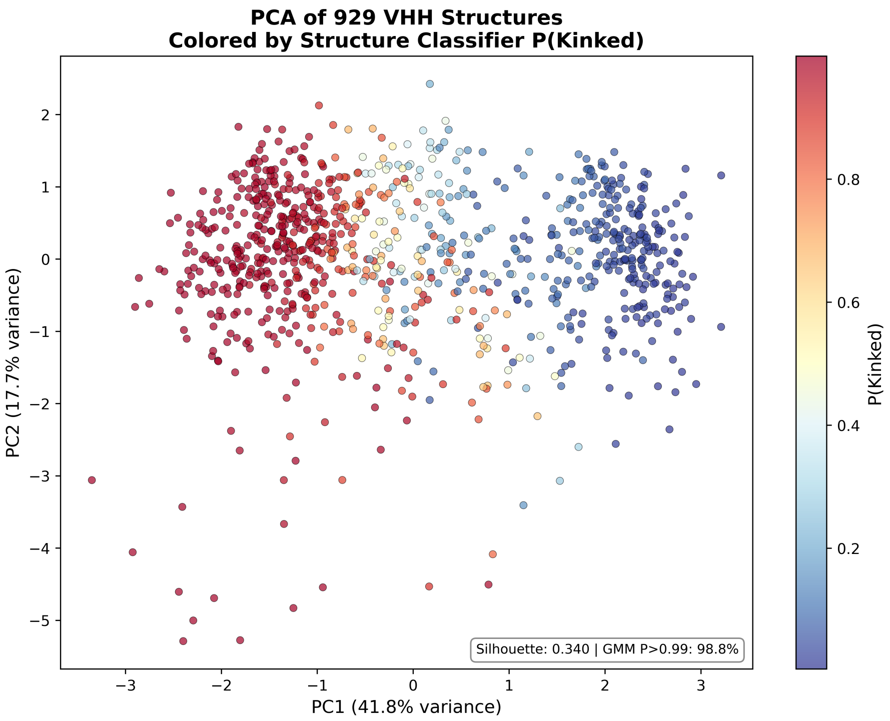

Figure S2: Principal Component Analysis (PCA) of 929 nanobody structures based on six structural features: N- and C-terminal CDR3 backbone angles (α_N, τ_N, α_C, τ_C), contact density (CDR3-FR2 contacts normalized by CDR3 length), and relative solvent accessibility of key FR2 positions (AHo 44 and 54). Points are coloured by the predicted probability of kinked conformation P(Kinked) from a logistic regression classifier trained on 100 expert-labelled structures from the training set. The two principal components explain 59.5% of the total variance (PC1: 41.8%, PC2: 17.7%). Separation between kinked (red, high P(Kinked)) and extended (blue, low P(Kinked)) conformations is observed, with a small population of intermediate structures in the transition zone. The moderate silhouette score (0.340) reflects the continuum between kinked and extended conformations, where sharp boundaries are not expected. However, Gaussian Mixture Model (GMM) analysis demonstrates that 98.8% of structures can be assigned to one of two clusters with confidence >0.99, indicating that the six-dimensional feature space provides discriminative power despite geometric overlap in the 2D projection.

bioRxiv preprint doi: https://doi.org/10.64898/2026.02.13.705647; this version posted February 13, 2026. The copyright holder for this preprint (which was not certified by peer review) is the author/funder, who has granted bioRxiv a license to display the preprint in perpetuity. It is made available under aCC-BY 4.0 International license.

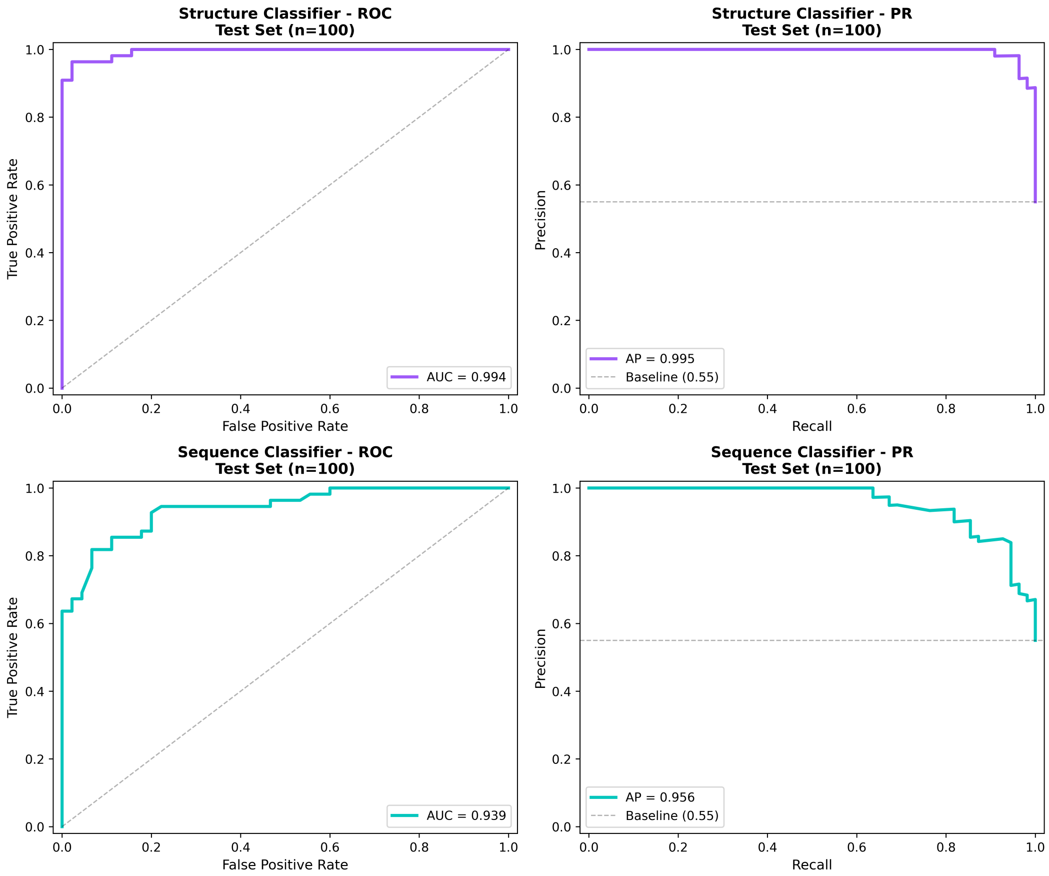

Figure S3: Performance evaluation of NbFrame classifiers on 100 held-out nanobody structures (PDB IDs starting with "9") with manual labels as ground truth. Top row: Structure-based classifier using six geometric features (N- and C-terminal CDR3 backbone angles, CDR3-FR2 contact density, and FR2 solvent accessibility at key positions). Bottom row: Sequence-based classifier using 20 framework hallmark features from 10 positions. Left column: Receiver Operating Characteristic (ROC) curves showing true positive rate versus false positive rate; dashed diagonal indicates random classification. Right column: Precision-Recall (PR) curves; dashed horizontal line indicates the baseline precision equal to the proportion of kinked structures in the test set (0.55). The structure classifier achieves near-perfect discrimination (ROC-AUC = 0.994, AP = 0.995), while the sequence classifier, using only amino acid sequence information, achieves strong performance (ROC-AUC = 0.939, AP = 0.956).

bioRxiv preprint doi: https://doi.org/10.64898/2026.02.13.705647; this version posted February 13, 2026. The copyright holder for this preprint (which was not certified by peer review) is the author/funder, who has granted bioRxiv a license to display the preprint in perpetuity. It is made available under aCC-BY 4.0 International license.

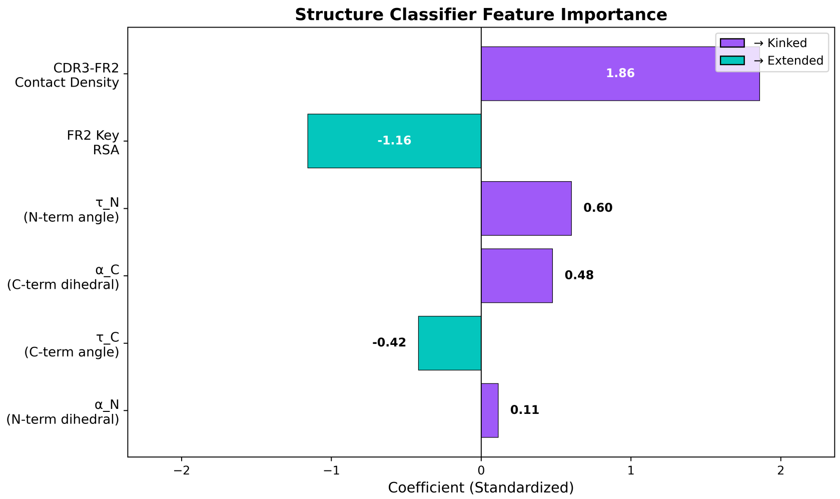

Figure S4: Structure classifier feature importance. Standardized logistic regression coefficients for the six structural features used in binary classification. Positive coefficients (purple) indicate features associated with kinked conformations, while negative coefficients (cyan) indicate features associated with extended conformations. CDR3-FR2 contact density (contacts normalized by CDR3 length at FR2 positions 44-55) is the strongest predictor of kinked conformation (coefficient: +1.86), followed by FR2 key position relative solvent accessibility (RSA at positions 44 and 54, coefficient: -1.16), where lower RSA indicates buried FR2 residues characteristic of kinked structures. The four backbone geometry features (τ_N, α_C, τ_C, α_N) contribute less to classification (coefficients: +0.60, +0.48, -0.42, +0.11), indicating that CDR3-FR2 packing interactions are more discriminative than CDR3 geometry alone. The trained classifier achieves ROC-AUC of 0.994 and 94% accuracy on the test set (n=100).

bioRxiv preprint doi: https://doi.org/10.64898/2026.02.13.705647; this version posted February 13, 2026. The copyright holder for this preprint (which was not certified by peer review) is the author/funder, who has granted bioRxiv a license to display the preprint in perpetuity. It is made available under aCC-BY 4.0 International license.

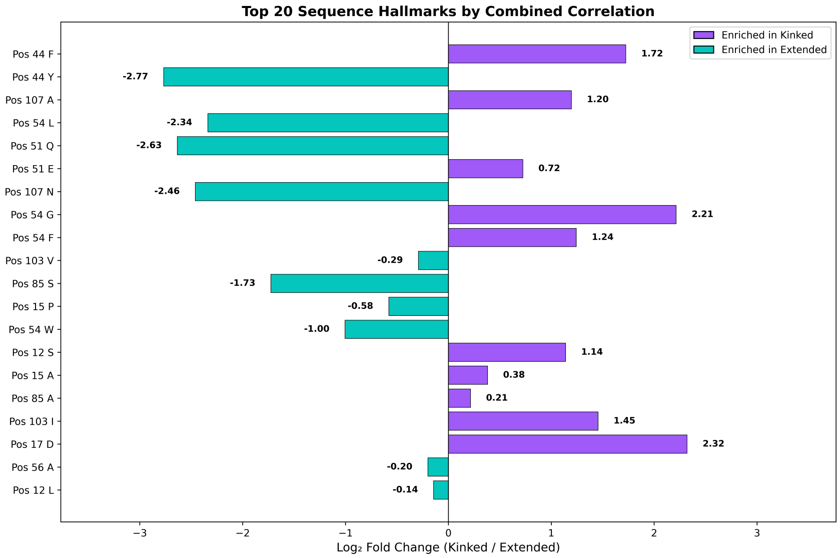

Figure S5: Top 20 sequence hallmarks for kinked/extended classification (AHo numbering). Log_2 fold change (Kinked/Extended) for position-specific amino acid features selected by combined correlation with both binary labels and structure classifier predictions. Positive values (purple) indicate amino acids enriched in kinked conformations, while negative values (cyan) indicate enrichment in extended conformations. Hallmarks are drawn from framework regions and the HCDR3 N-terminal anchor position, concentrated at the CDR3-framework interface: FR2 positions 44, 51, 54, 56 (9 features), FR3 positions 85 and 103 (4 features), FR1 positions 12, 15, 17 (5 features), and position 107 at the HCDR3 N-terminal stem (2 features). Position 107, immediately C-terminal to the conserved Cys106, anchors the HCDR3 loop to the framework; unlike hypervariable CDR positions, it shows limited but functionally meaningful sequence variation associated with loop conformation. The strongest discriminative features are at FR2 position 44, where phenylalanine (F, $\log_2$FC: +1.72) strongly predicts kinked conformation and tyrosine (Y, $\log_2$FC: -2.77) predicts extended conformation. Other key hallmarks include position 107 alanine (+1.20, kinked), position 54 glycine (+2.21, kinked), and position 51 glutamine (-2.63, extended). The sequence classifier trained on these 20 hallmarks achieves ROC-AUC of 0.939 and 86% accuracy on the test set ($n = 100$), with strong correlation to the structure classifier (Pearson r=0.812).

bioRxiv preprint doi: https://doi.org/10.64898/2026.02.13.705647; this version posted February 13, 2026. The copyright holder for this preprint (which was not certified by peer review) is the author/funder, who has granted bioRxiv a license to display the preprint in perpetuity. It is made available under aCC-BY 4.0 International license.

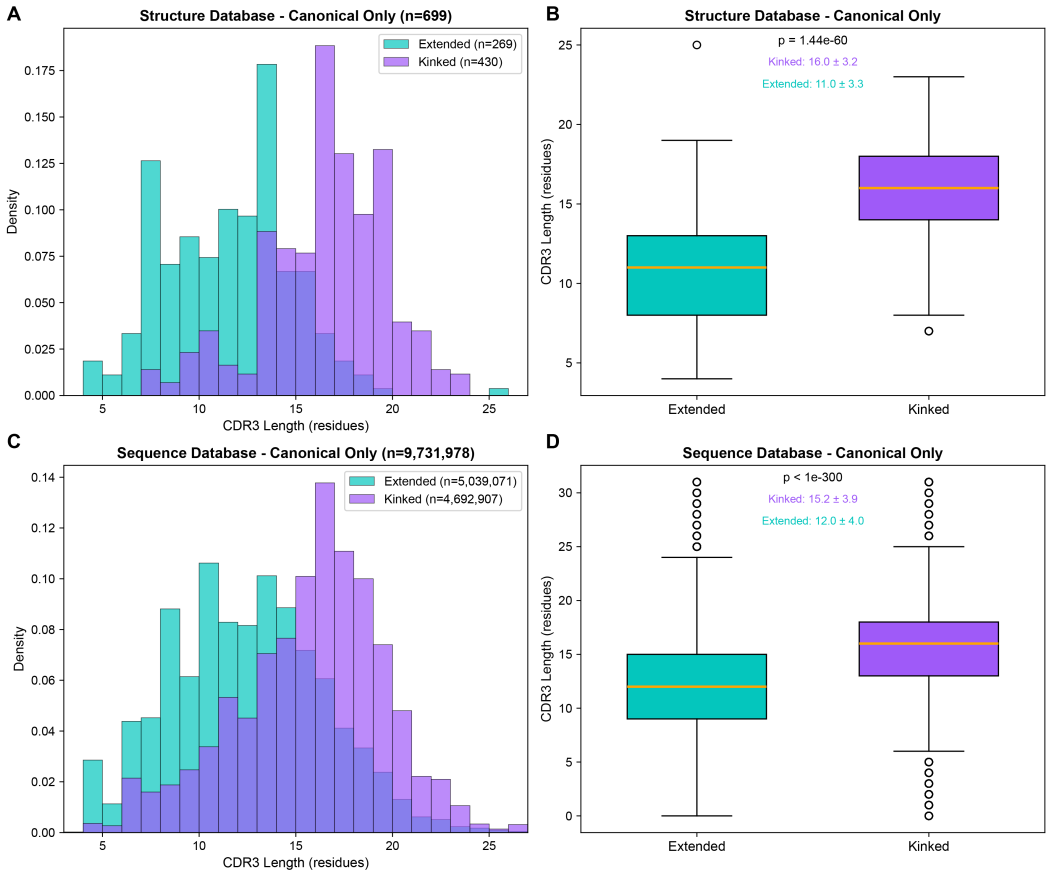

Figure S6: Relationship between CDR3 length and predicted conformation in nanobodies with canonical disulphide bonds. Analysis was restricted to sequences containing exactly two cysteine residues to exclude nanobodies with non-canonical disulphide bonds, which tend to have longer CDR3 loops and are predominantly kinked. Only confident predictions were included, excluding sequences in the classifier uncertainty zones. **(A-B):** Structure database ($n = 699$). Predicted kinked conformations (purple, $n = 430$) have significantly longer CDR3 loops (mean 16.0 ± 3.2 residues) compared to predicted extended conformations (teal, $n = 269$; mean 11.0 ± 3.3 residues; Mann-Whitney U test, p = 1.44×10^{-60}). **(C-D):** Sequence database ($n = 9,731,978$). The same trend is observed at scale, with predicted kinked sequences ($n = 4,692,907$) having longer CDR3 loops (mean 15.2 ± 3.9 residues) than predicted extended sequences ($n = 5,039,071$; mean 12.0 ± 4.0 residues; $p < 10^{-300}$). Default classifiers thresholds were used: structure classifier (extended: P<0.25, kinked: P>0.55); sequence classifier (extended: P<0.40, kinked: P>0.70).

bioRxiv preprint doi: https://doi.org/10.64898/2026.02.13.705647; this version posted February 13, 2026. The copyright holder for this preprint (which was not certified by peer review) is the author/funder, who has granted bioRxiv a license to display the preprint in perpetuity. It is made available under aCC-BY 4.0 International license.

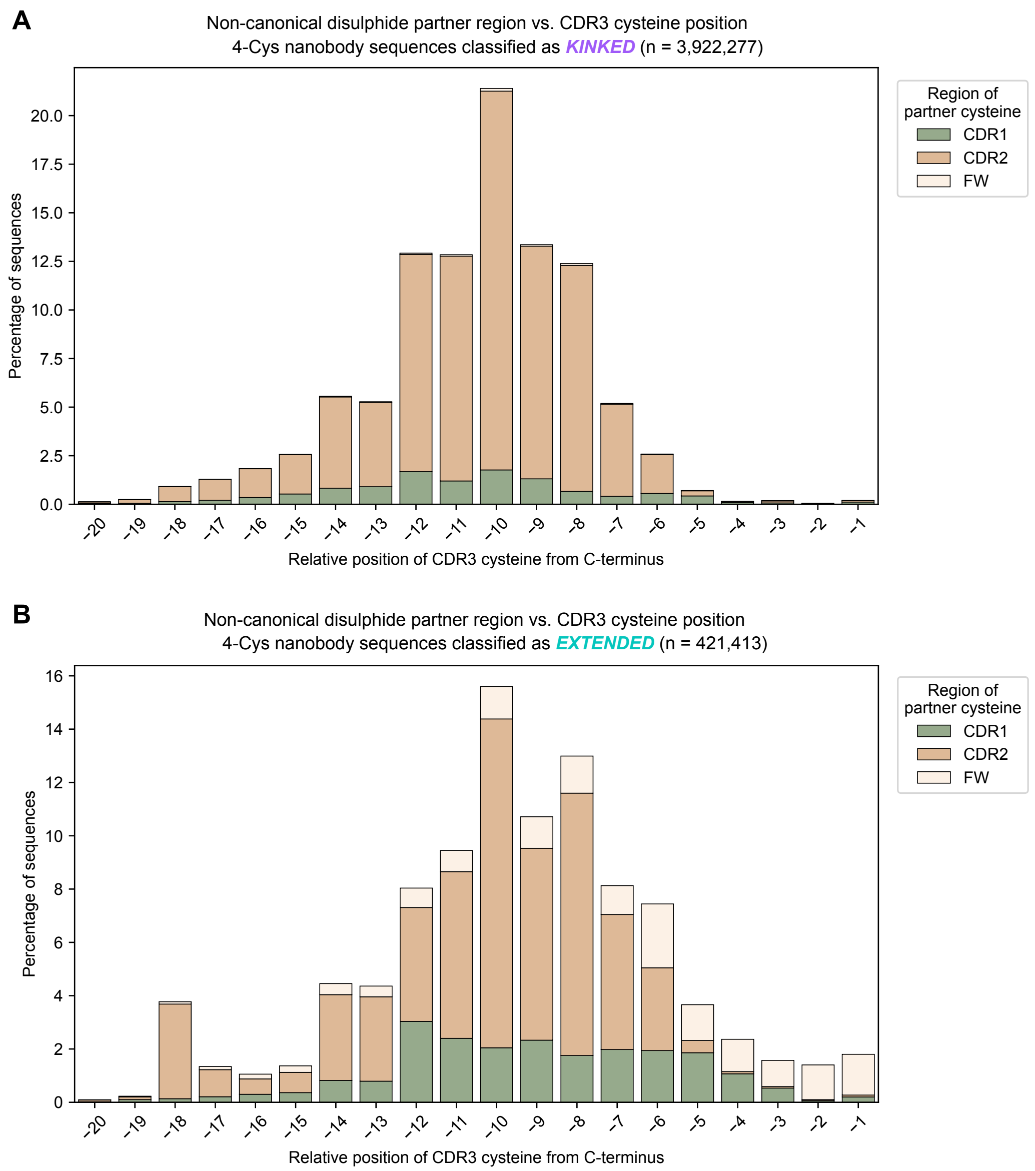

Figure S7: Structural region of the putative non-canonical disulphide partner cysteine as a function of CDR3 cysteine position. Analysis was restricted to sequences containing exactly four cysteine residues: the canonical disulphide pair (AHo positions 23 and 106), exactly one additional cysteine within CDR3 (AHo positions 108–138), and one partner cysteine elsewhere in the sequence, presumably forming a non-canonical disulphide bond with the CDR3 cysteine. The x-axis indicates the relative position of the CDR3 cysteine from the C-terminus of CDR3 (AHo position 138 $= -1$), counting only non-gap residues. Stacked bars are coloured by the structural region of the partner cysteine: CDR1 (AHo positions 27–42, green), CDR2 (AHo positions 57–69, tan), or framework (all other non-CDR3 positions, cream). **(A)** Sequences predicted as kinked ($n = 3,922,277$). The partner cysteine is overwhelmingly located in CDR2, with a minor contribution from CDR1 and nearly no framework partners, consistent with the well-characterised CDR2–CDR3 non-canonical disulphide bond that stabilises the kinked CDR3 conformation. The CDR3 cysteine position shows a pronounced peak at approximately -10 from the C-terminus. **(B)** Sequences predicted as extended ($n = 421,413$). CDR2 remains the predominant partner region but with substantially greater contributions from both CDR1 and framework compared to kinked sequences. The CDR3 cysteine positions are more broadly distributed across the CDR3 loop, suggesting more diverse non-canonical disulphide bond geometries in the extended conformation. Default sequence classifier thresholds were used (extended: P<0.40, kinked: P>0.70).

bioRxiv preprint doi: https://doi.org/10.64898/2026.02.13.705647; this version posted February 13, 2026. The copyright holder for this preprint (which was not certified by peer review) is the author/funder, who has granted bioRxiv a license to display the preprint in perpetuity. It is made available under aCC-BY 4.0 International license.

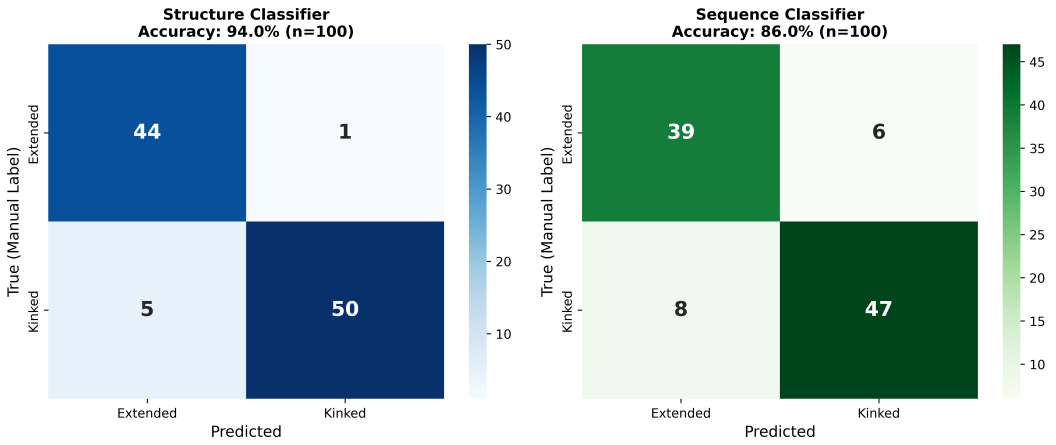

Figure S8: Test set confusion matrices for both classifiers. Confusion matrices showing classification performance on 100 held-out test structures (PDB IDs starting with '9'). **Left:** Structure classifier achieves 94% accuracy with high precision for both classes. True negatives (Extended correctly classified): 44/45 (97.8%). True positives (Kinked correctly classified): 50/55 (90.9%). Only 6 total misclassifications: 1 false positive and 5 false negatives, all occurring in structures with intermediate predicted probabilities ($0.3 < P < 0.7$). **Right:** Sequence classifier achieves 86% accuracy with balanced performance. True negatives: 39/45 (86.7%). True positives: 47/55 (85.5%). Total misclassifications: 14 (6 false positives, 8 false negatives). The sequence classifier's error distribution is more symmetric, reflecting the greater challenge of predicting 3D conformation from sequence information alone. Both classifiers show balanced precision and recall, with no systematic bias toward either class.

Model Performance Comparison

Metric	Structure Classifier	Sequence Classifier
Training samples	100 (expert)	829 (soft labels)
Features	6 structural	20 hallmarks
CV ROC-AUC	0.961	0.855
CV Accuracy	90.0%	81.9%
Test ROC-AUC	0.994	0.939
Test Accuracy	94.0%	86.0%
Test Pearson r	—	0.812
Agreement vs Struct	—	86.0%

Figure S9: NbFrame classifier performance comparison. Summary of key performance metrics for the NbFrame structure and sequence classifiers evaluated on the same 100-structure test set (PDB IDs starting with '9'). The structure classifier was trained on 100 manually labelled structures using 6 structural features (CDR3 backbone angles, CDR3-FR2 contact density, and FR2 key position RSA), achieving cross-validation ROC-AUC of 0.961 (90% accuracy) and test set ROC-AUC of 0.994 (94% accuracy). The sequence classifier was trained on all 829 training structures with soft probability labels from the structure classifier, using 20 framework sequence hallmarks selected by combined correlation. It achieves cross-validation ROC-AUC of 0.855 (81.9% accuracy) and test set ROC-AUC of 0.939 (86% accuracy). The sequence classifier shows strong correlation with the structure classifier (Pearson r=0.812) and 86% classification agreement on the test set. Both classifiers demonstrate excellent generalization, with test performance meeting or exceeding cross-validation performance. The structure classifier's superior performance reflects direct access to 3D structural information, while the sequence classifier's strong performance demonstrates that CDR3 conformation can be predicted from framework sequence hallmarks alone with high confidence.

bioRxiv preprint doi: https://doi.org/10.64898/2026.02.13.705647; this version posted February 13, 2026. The copyright holder for this preprint (which was not certified by peer review) is the author/funder, who has granted bioRxiv a license to display the preprint in perpetuity. It is made available under aCC-BY 4.0 International license.

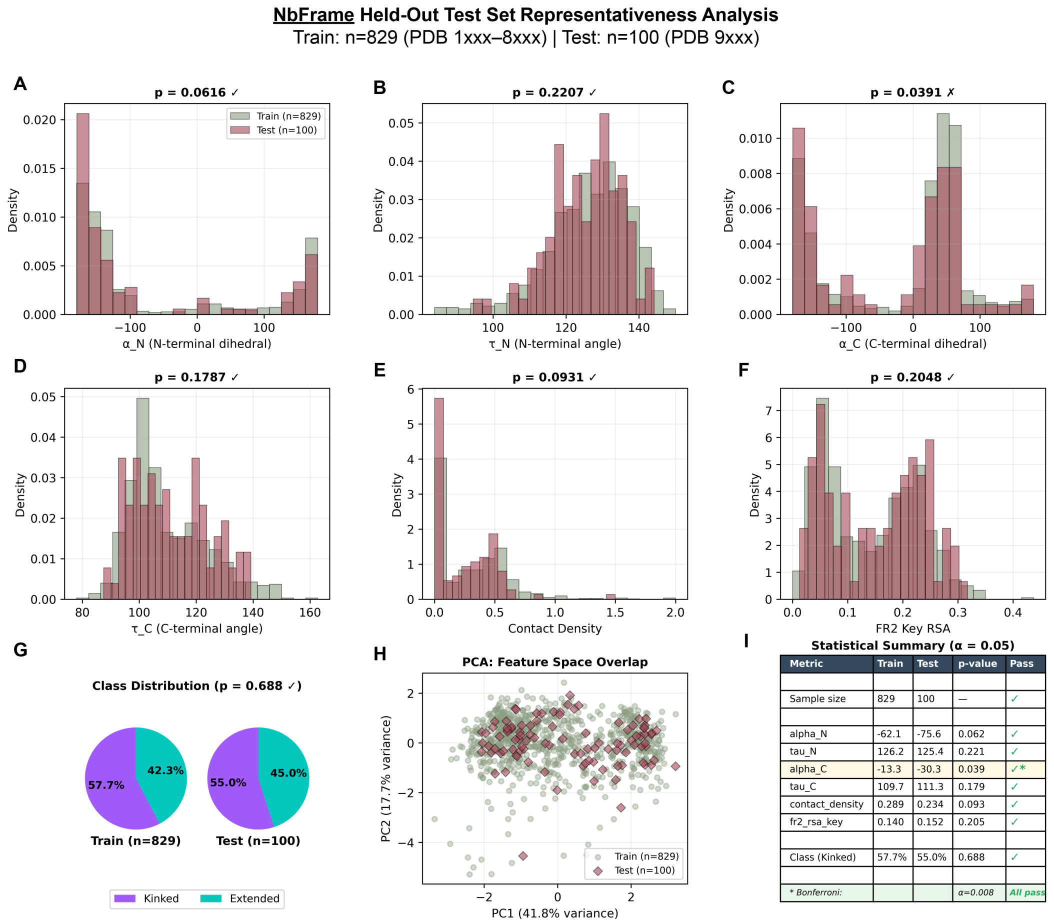

Figure S10: NbFrame Held-out test set representativeness analysis. Statistical comparison between NbFrame training ($n = 829$, PDB 1xxx–8xxx, green) and test ($n = 100$, PDB 9xxx, red) sets to verify unbiased temporal split. **(A–F)** Distribution comparisons for all six structural features using overlapping histograms with Mann-Whitney U test p-values. Five of six features show no significant difference (p>0.05): alpha_N (p=0.062), tau_N (p=0.221), tau_C (p=0.179), contact_density (p=0.093), and fr2_rsa_key (p=0.205). The alpha_C feature shows marginal significance (p=0.039) at the uncorrected threshold; however, when testing multiple features ($n = 6$), the Bonferroni-corrected significance threshold is α=0.05/6=0.008, and all features including alpha_C pass this more stringent criterion. This correction accounts for the increased risk of false positives when performing multiple statistical tests simultaneously. Additionally, alpha_C has minimal impact on classification (coefficient rank 4/6, standardized coefficient +0.477) with the model primarily driven by contact_density (+1.859) and fr2_rsa_key (-1.158). **(G)** Class distribution comparison via side-by-side pie charts shows similar proportions of Kinked structures in training (57.7%, 478/829) and test (55.0%, 55/100) sets (chi-squared test p=0.688), confirming balanced class representation. **(H)** Principal component analysis of the six-dimensional feature space demonstrates substantial overlap between training (green circles) and test (red diamonds) distributions. PC1 and PC2 explain 41.8% and 10.7% of variance respectively, with test structures distributed throughout the training feature space rather than clustered separately, indicating the test set samples from the same underlying distribution. **(I)** Statistical summary table showing all features with green checkmarks, including alpha_C marked with an asterisk (*) to denote marginal p-value at the uncorrected threshold. The bottom row notes that with Bonferroni correction (α=0.008), all features pass the representativeness criterion. Overall, the held-out test set is representative of the training set across structural features, class proportions, and feature space geometry, validating the temporal split strategy for unbiased model evaluation and supporting generalization of classifier performance to unseen structures.

bioRxiv preprint doi: https://doi.org/10.64898/2026.02.13.705647; this version posted February 13, 2026. The copyright holder for this preprint (which was not certified by peer review) is the author/funder, who has granted bioRxiv a license to display the preprint in perpetuity. It is made available under aCC-BY 4.0 International license.

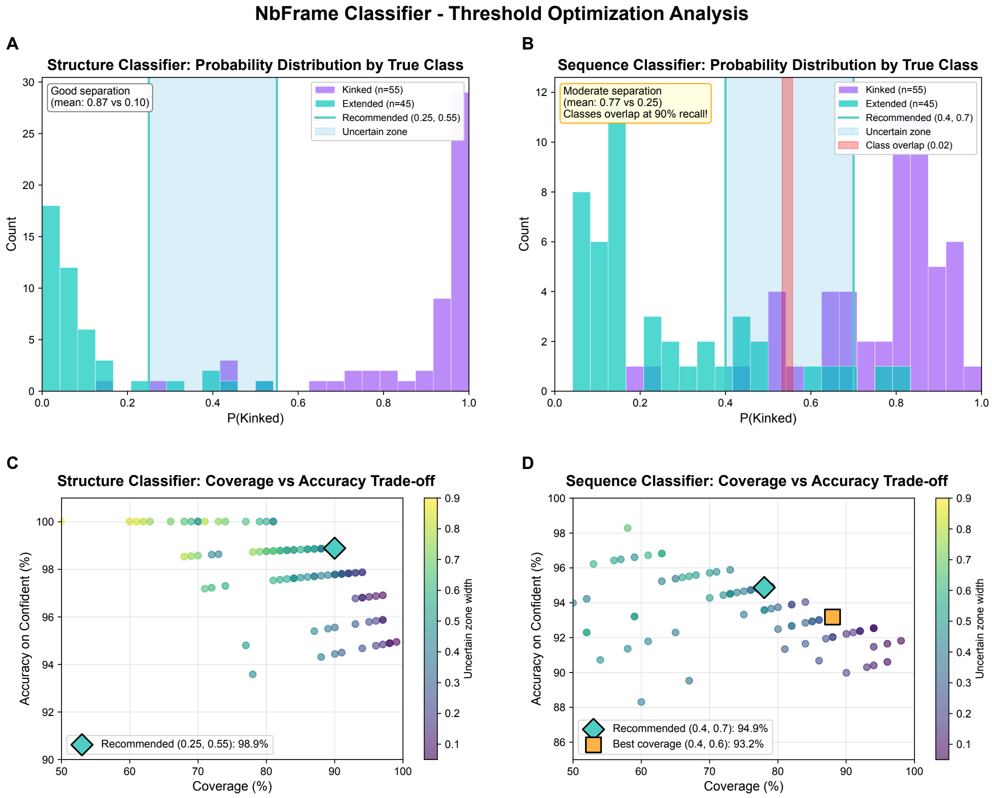

E

Recommended Threshold Summary

Classifier	Thresholds	Coverage	Accuracy
Structure	(0.25, 0.55)	90%	98.9%
Sequence	(0.4, 0.7)	78%	94.9%
Sequence (alternative)	(0.4, 0.6)	88%	93.2%

Figure S11: NbFrame classifier threshold optimization analysis. Systematic evaluation of classification thresholds for the structure-based and sequence-based classifiers on the held-out test set ($n = 100$ manually labelled structures: 55 Kinked, 45 Extended). **(A)** Structure classifier probability distribution by true class shows excellent separation between Kinked (purple, mean P(Kinked)=0.87) and Extended (teal, mean P(Kinked)=0.10) structures, with a class mean gap of 0.77. Recommended thresholds (0.25, 0.55) define the uncertain zone (blue shading) where predictions are withheld due to ambiguity. **(B)** Sequence classifier probability distribution shows moderate separation between classes (Kinked mean=0.77, Extended mean=0.25), with notable class overlap (red shading) at the 90% recall threshold, reflecting the fundamental limitation of sequence-only prediction compared to structure-based classification. Recommended thresholds (0.4, 0.7) balance accuracy and coverage. **(C)** Structure classifier coverage versus accuracy trade-off across all threshold combinations tested via grid search. Points are colored by uncertain zone width. The recommended thresholds (0.25, 0.55; diamond marker) achieve 90% coverage with 98.9% accuracy on confident predictions, representing an optimal balance between classification certainty and sample utilization. **(D)** Sequence classifier coverage versus accuracy trade-off. The recommended thresholds (0.4, 0.7; diamond marker) achieve 78% coverage with 94.9% accuracy. An alternative configuration (0.4, 0.6; square marker) prioritizes coverage (88%) with slightly reduced accuracy (93.2%) for applications where maximum sample utilization is preferred. **(E)** Summary Table: Recommended (default) threshold configurations for both classifiers. The structure classifier achieves near-perfect accuracy (98.9%) while maintaining high coverage (90%). The sequence classifier offers two operating points: the default (0.4, 0.7) optimizes accuracy at 94.9% with 78% coverage, while the alternative (0.4, 0.6) maximizes coverage at 88% with 93.2% accuracy.

bioRxiv preprint doi: https://doi.org/10.64898/2026.02.13.705647; this version posted February 13, 2026. The copyright holder for this preprint (which was not certified by peer review) is the author/funder, who has granted bioRxiv a license to display the preprint in perpetuity. It is made available under aCC-BY 4.0 International license.

Table S5: Coordinate accuracy and feature recovery on the NbForge test set. Mean Backbone (CA, N, CO, O) RMSDs are reported as mean ± standard error (SE) in Å (n = 47) after framework superimposition. Kinked/extended (K/E) blueprint recovery is computed over all test structures (see Methods), while non-canonical disulphide bond (NCDB) recovery is computed over the subset containing a NCDB (n = 15), see Methods.

Model	All	FR	HCDR1	HCDR2	HCDR3	K/E rec. (%)	NCDB rec. (%)
AF3	1.47 ± 0.10	0.69 ± 0.04	1.57 ± 0.15	1.06 ± 0.09	3.25 ± 0.30	93.6	93.3
Boltz1	1.56 ± 0.10	0.69 ± 0.04	1.66 ± 0.16	1.21 ± 0.13	3.39 ± 0.30	89.4	73.0
NBB2	1.78 ± 0.08	0.81 ± 0.04	1.87 ± 0.12	1.35 ± 0.12	4.03 ± 0.25	80.9	26.7
NbForge	1.57 ± 0.07	0.79 ± 0.03	1.65 ± 0.11	1.36 ± 0.11	3.40 ± 0.24	91.5	86.7

Table S6: Mean Backbone coordinate accuracy (RMSD, Å) stratified by presence of a non-canonical disulphide bond (NCDB n = 15) or by its absence (no NCDB n = 32). RMSDs are calculated for all backbone heavy atoms following framework superimposition and reported as median with bootstrap 95% confidence intervals.

NCDB	Region	AF3	Boltz1	NBB2	NbForge
no NCDB	FR	0.71 ± 0.05	0.72 ± 0.05	0.83 ± 0.05	0.82 ± 0.05
	HCDR1	1.49 ± 0.19	1.48 ± 0.18	1.83 ± 0.16	1.64 ± 0.15
	HCDR2	0.98 ± 0.11	1.09 ± 0.13	1.22 ± 0.12	1.31 ± 0.13
	HCDR3	3.15 ± 0.41	3.30 ± 0.40	4.11 ± 0.33	3.47 ± 0.33
NCDB	FR	0.65 ± 0.04	0.63 ± 0.05	0.77 ± 0.04	0.73 ± 0.04
	HCDR1	1.60 ± 0.27	1.90 ± 0.28	1.94 ± 0.19	1.63 ± 0.16
	HCDR2	1.24 ± 0.16	1.52 ± 0.31	1.65 ± 0.24	1.50 ± 0.22
	HCDR3	3.39 ± 0.45	3.50 ± 0.45	3.92 ± 0.39	3.23 ± 0.27

Table S7: Coordinate accuracy and feature recovery on the NbForge test set. Mean Backbone (CA, N, CO, O) RMSDs are reported as mean ± standard error (SE) in Å (n = 47) after framework superimposition. Kinked/extended (K/E) blueprint recovery is computed over all test structures (see Methods), while non-canonical disulphide bond (NCDB) recovery is computed over the subset containing a NCDB (n = 15), see Methods. NbForge models without OpenMM relaxation are used in these calculations.

Model	All	FR	HCDR1	HCDR2	HCDR3	K/E rec. (%)	NCDB rec. (%)
AF3	1.47 ± 0.10	0.69 ± 0.04	1.57 ± 0.15	1.06 ± 0.09	3.25 ± 0.30	93.6	93.3
Boltz1	1.56 ± 0.10	0.69 ± 0.04	1.66 ± 0.16	1.21 ± 0.13	3.39 ± 0.30	89.4	73.0
NBB2	1.78 ± 0.08	0.81 ± 0.04	1.87 ± 0.12	1.35 ± 0.12	4.03 ± 0.25	80.9	26.7
NbForge	1.57 ± 0.07	0.79 ± 0.03	1.65 ± 0.11	1.35 ± 0.11	3.38 ± 0.23	89.4	80.0

Table S8: Coordinate accuracy and feature recovery on the NbForge test set. Backbone (CA, N, CO, O) RMSDs are reported as median with bootstrap 95% confidence intervals (CI) in Å (n = 47) after framework superimposition. Confidence intervals were estimated using non-parametric bootstrap resampling with 10,000 resamples. Kinked/extended (K/E) correctness is computed over all test structures, while non-canonical disulphide bond (NCDB) recovery is computed over the subset containing a non-canonical disulphide bond (n = 15), see Methods. NbForge models without OpenMM relaxation are used in these calculations.

Metric	AF3	Boltz1	NBB2	NbForge
All	1.39 [1.05, 1.53]	1.52 [1.23, 1.76]	1.62 [1.51, 1.91]	1.47 [1.34, 1.63]
FR	0.70 [0.57, 0.74]	0.65 [0.58, 0.73]	0.79 [0.67, 0.85]	0.73 [0.69, 0.78]
HCDR1	1.19 [1.03, 1.50]	1.28 [1.02, 1.63]	1.72 [1.53, 2.17]	1.57 [1.36, 1.77]
HCDR2	0.80 [0.67, 1.26]	0.89 [0.63, 1.33]	1.17 [0.94, 1.44]	1.17 [1.02, 1.43]
HCDR3	2.95 [2.30, 3.66]	3.39 [2.47, 3.74]	3.45 [2.99, 4.53]	2.97 [2.72, 3.63]
K/E recovery (%)	93.6	89.4	80.9	89.4
NCDB recovery (%)	93.3	73.0	26.7	80.0
